# Supplementary material for: In-chip critical plasma seeds for laser writing of reconfigurable silicon photonics systems
Source: Nat Commun. 2025 Jul 22;16:6733. doi: 10.1038/s41467-025-61983-9 (PMC12284106; doi:10.1038/s41467-025-61983-9)
Supplement: Supplementary file 1 — Supplementary Information [file 41467_2025_61983_MOESM1_ESM.pdf]

Supplementary information

In-chip critical plasma seeds for laser writing of reconfigurable silicon photonics systems

Andong Wang[1,2], Amlan Das[1], Vladimir Yu Fedorov[3,4], Pol Sopeña[1], Stelios Tzortzakis[3,5,6] and David Grojo[1,#]

[1]*Aix-Marseille University, CNRS, LP3 UMR 7341, F-13288, Marseille, France*
[2] *Laser Micro/Nano Fabrication Laboratory, School of Mechanical Engineering, Beijing Institute of Technology, Beijing 100081, China*
[3]*Science Program, Texas A&M University at Qatar, P.O. Box 23874 Doha, Qatar*
[4]*P. N. Lebedev Physical Institute of the Russian Academy of Sciences*
[5]*Institute of Electronic Structure and Laser (IESL), Foundation for Research and Technology - Hellas (FORTH), P.O. Box 1527, GR-71110 Heraklion, Greece*
[6]*Materials Science and Technology Department, University of Crete, 71003, Heraklion, Greece*

[#] *Email: david.grojo@cnrs.fr*

Contents

Note 1: Experimental Setup 3
Note 2: Conditions for writing with sub-diffraction-limit resolution 5
2.1 Measurements of modification sizes for different seeding energy conditions 5
2.2 Performance comparisons for three-dimensional (3D) writing 5
Note 3. Drude model for plasma density estimations 8
Note 4. Interaction simulations 9
4.1 Simulation methods 9
4.2 Influence of the pre-plasma on the picosecond pulse interactions 10
4.3 Potential propagation effects resulting from plasma seeding 13
4.4 Simulated modification positions for different plasma sizes 13
Note 5: Methods for relative spot motions 15
5.1 Lateral shift (in X) 15
5.2 Longitudinal shift (in Z) 15
Note 6. Characterizations of modifications 16
6.1 Raman Spectroscopy 16
6.2 Transmission electron microscopy (TEM) 16
Note 7. Laser erasing of modifications 20
7.1 Setup and Method 20
7.2 Laser conditions for efficient erasing 21
7.3 Inscription of QR codes inside Si 25
Note 8. Phase-plate fabrication, measurement and applications 27
8.1 Methods for quantitative phase infrared imaging 27
8.2 Phase measurements 28
8.3 Beam shaping demonstrations 29
References 32

Note 1: Experimental Setup

This note describes the details of the experimental configurations used for this work as schematically depicted in Fig. S1-1.

Delivery of multi-timescale pulses. We use a femtosecond laser source (Pharos, Light-conversion) generating 500-μJ pulses at 1030-nm central wavelength. Then, the beam is converted to 1550 nm by using an OPA (Orpheus, Light-conversion) delivering pulses of sub-190-fs duration (FWHM as measured by autocorrelation) with high temporal contrast[1]. To generate the picosecond writing pulse, we split the beam to inject a portion in a stretcher arrangement with two Littrow gratings introducing negative group velocity dispersion to the original laser pulses. The details of the configuration are discussed elsewhere[2].The distance between the gratings (see stage 2) can be changed to tune the duration of the stretched pulses from 4 to 21 ps. After passing through a delay line, the stretched pulses are then inserted into the path of the main femtosecond laser beam (see stage 1). For the purpose of modification erasing, we also synchronize a 10-μJ class nanosecond laser source (MWTech, PFL-1550) which generates pulses at 1550-nm wavelength. The pulse duration is 5 ns (FWHM). It is externally triggered using the femtosecond source as clock and the delay between the nanosecond and femtosecond pulses are changed (±100 ns) with 25 ps precision by simply using a digital delay generator (Standford DG645). For the experiments presented in this paper, the nanosecond laser is used independently to the femtosecond and picosecond beams (both blocked by a mechanical shutter) but we take benefit of the configuration to have the same control method for all pulses. Another important detail is the use of a telescope in the beam path of the nanosecond laser (not shown) to obtain beams of identical size.

Synchronization of the pulses and focal-spot overlap. The temporal delay between the pulses is measured by a self-assembled long-scan auto-correlator (Newport). The collinearity of the three beams after recombination using thin-film beam splitters is made with the aid of two irises with >1 m separation. Then, for interaction studies, the three-component beam is tightly focused using a 0.85-NA microscope objective (Olympus LCPLN-IR 100×) with spherical aberration compensation. A high-resolution imaging system is also used to guarantee nearly perfect spatial overlap of the focused beams from the three components.

Laser-induced modifications. For the writing experiments, the prepared pulse-sequences are focused 300 μm below the surface of Si samples. The samples are 1-mm thick intrinsic Si crystals with 100 (±5∘) orientation. The lasers are operated at 1-kHz repetition rate and a fresh region of the sample is systematically irradiated and observed with an infrared microscopy system. The latter consists in the assembly of a microscope objective, a tube lens and an InGaAs array detector behind the sample for backside *in situ* detection of the potential micro-modifications. For each double-pulse delay condition (synchronized femtosecond and picosecond pulses), the bulk modification energy threshold (when modification is achievable) is estimated by irradiating fresh regions of the sample with gradually decreasing pulse energies until no modification is observed. Then, the precision for threshold determination is directly depending on the energy steps used in each procedure.

Pump-probe transmittance microscopy. To observe the dynamics of the plasma, probe pulses from the same laser system are precisely delayed by stage 3 and used to illuminate the plasma. An infrared microscopy imaging system (not shown in Fig. S1-1), consisting of a long working distance objective (Mitutoyo 20 , 0.40NA), a tube lens, and a camera (Raptor, OWL640), is added to observe laterally the laser interactions inside Si. Shadowgraphy images of the plasma are captured to calculate the plasma densities from the measured local transmittance in the images.

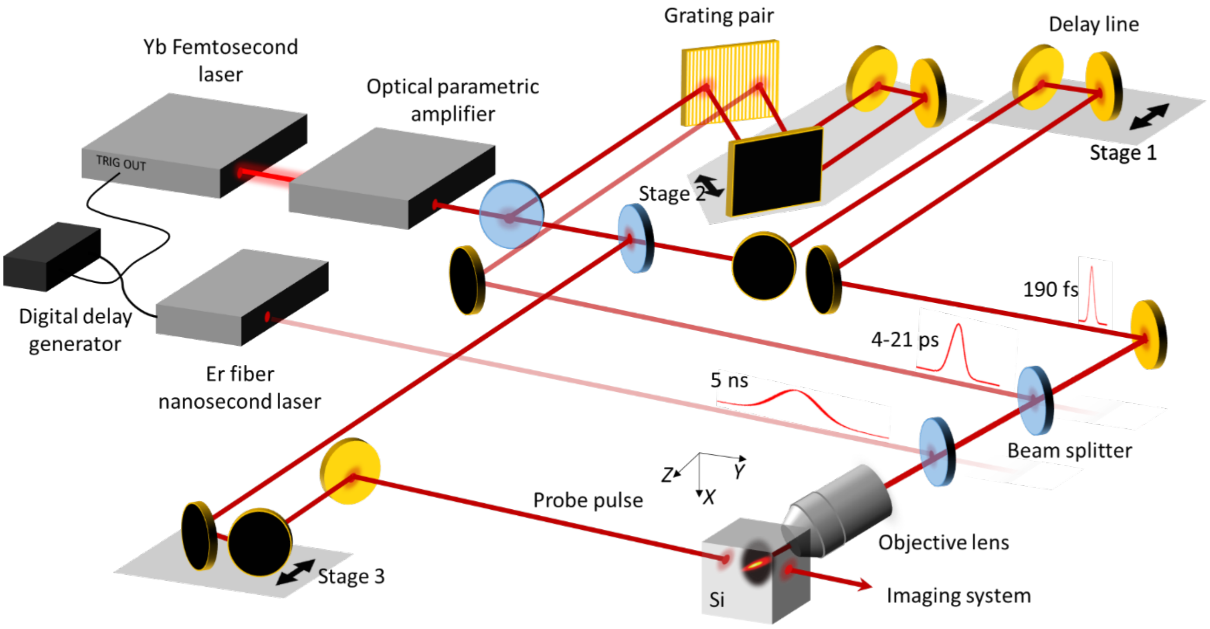

Fig. S1-1. Simplified schematic representation of the experimental setup for the multi-timescale writing and pump-probe studies.

Note 2: Conditions for writing with improved resolution

This note describes the range of experiments performed to assess the achievable writing precision and prepare performance demonstrations.

2.1 Measurements of modification sizes for different seeding energy conditions

The size of the produced modifications inside silicon with the plasma-seeding approach depends on both the energy of the femtosecond pre-pulse and the delayed picosecond writing pulse. Therefore, we use different pulse combinations to investigate how each parameter influences the results. Some produced features after 1000 pulses are shown in Fig. S2-1a as observed by infrared microscopy. The size of the observed features along X (lateral writing resolution) or Z (longitudinal writing resolution) axes are measured and plotted in Fig. S2-1b or Fig. S2-1c. The volume of the modification is derived by assuming the shape of the modifications as a spheroid.

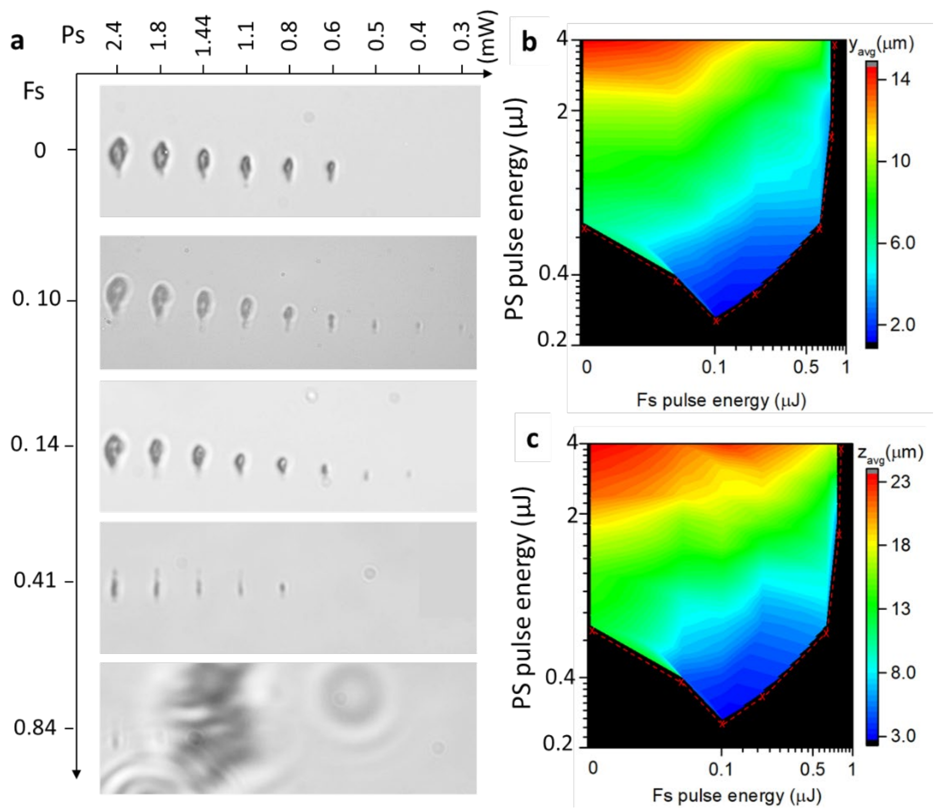

Fig. S2-1. Dimensions of the written spots produced by different combinations of femtosecond and picosecond pulses. a. Modification observations after irradiation with different pulse energy combinations. **b-c.** Measured sizes of the modifications, respectively laterally and longitudinally. Each measured value is an average of 3 measurements. The red crosses and dot-lines in the maps indicate tested conditions without modification. The difference with the beginning of the coloured region then corresponds to the error bar for the determination of threshold conditions.

2.2 Performance comparisons for three-dimensional (3D) writing

For comparisons, the transverse writing resolution and 3D writing precision are evaluated for the case of single picosecond pulses and with plasma seeded conditions (adding an optimum femtosecond laser pre-pulse). Fig. S2-2a. shows the transversely written structures using identical picosecond pulse conditions for both cases. The 'pure' picosecond case leads to discontinuities in the produced features while the plasma-seeded conditions lead to improved performances.

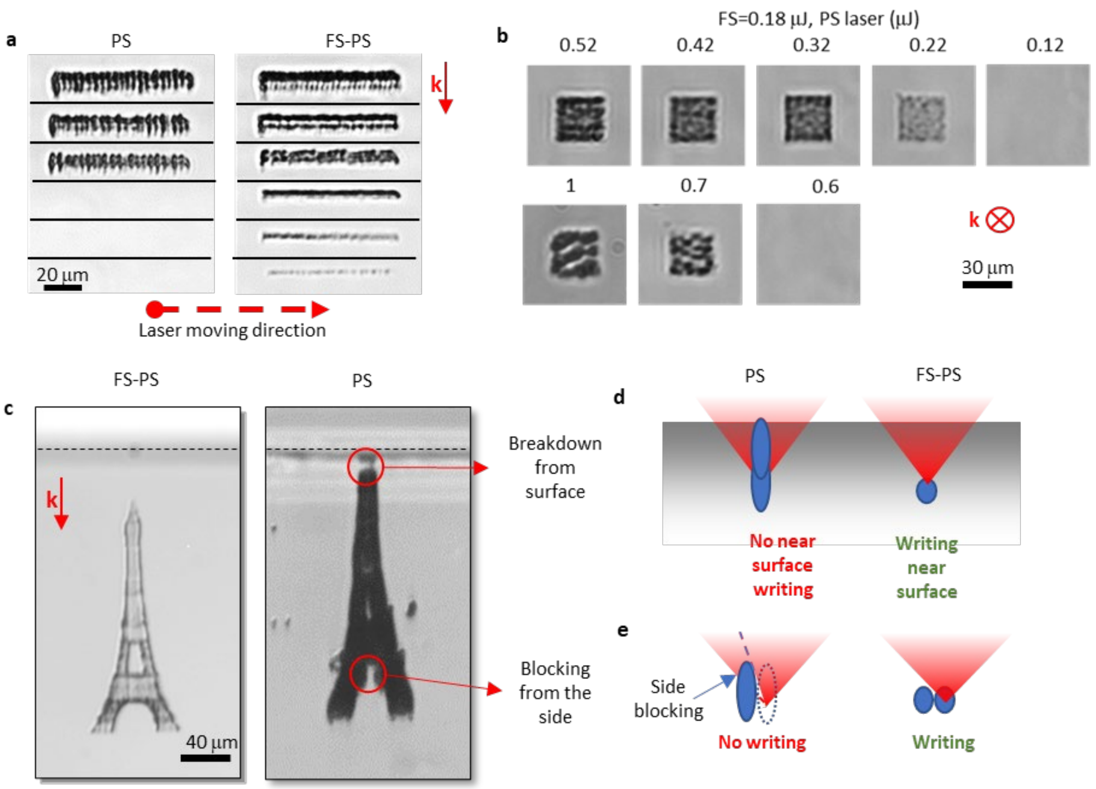

Fig. S2-2. Comparison between the writing results obtained by picosecond laser writing with and without plasma-seeded conditions. a. Transverse writing of lines. The writing speed is 10 µm/s and the repetition rate is 1 kHz. From bottom to top, the energy of the applied picosecond pulses varies and ranges from 0.3 to 0.8 µJ with a step size of 0.1 µJ. The femtosecond pre-pulse energy is 0.15 µJ. **b.** Transverse writing of a square. Each square consists of an assembly of spots with 1−µm separation. The total size is 30 µm by 30 µm. **c**. Tentative 3D writing of Eiffel towers for the best conditions with and without plasma-seeding. **d-e**. Illustrations of the reasons for the observed defects including surface damage and missing spots when using picosecond laser writing.

Fig. S2-2b. shows 2D transversely written structures after scanning procedures along the X and Y axes (the coordinate is shown in Fig. S1-1). The conclusion here is similar to the line writing case. Much smoother results can be obtained for plasma-seeded writing, especially when using a low picosecond pulse energy.

Fig. S2-2c compares 3D writing results. 3D Eiffel towers are written point-by-point, layer-by-layer from bottom to top with optimized conditions. Each voxel is created with 100 applied shots and the pitch between voxels is 2µm. An essential aspect was spherical aberration compensation. Based on the design shown in Fig. 2d of the main manuscript, the fabrication is done in three steps (corresponding to three distinct layer ranges). For each step at different depths, the spherical aberration compensation is manually readjusted with the correction collar of the microscope objective. The pulse energy is set to be 10% above the threshold (femtosecond pre-pulse energy 0.1-µJ) for writing as uniform as possible without complications introduced with overlapping spots. The written structures reveal less precision performance with pure PS writing.

The main noticeable imperfections with the pure picosecond writing method include surface breakdown and missing of parts in the written structure. The latter can be explained with schematics presented in Fig. S2-2d. First, because of the produced relatively large interaction voxels resulting from picosecond laser writing, the modifications tend to rapidly grow in the pre-focal region. For tentative writing very

close to the surface, this easily causes surface damage. Second, the large written voxels and the nature of the modifications obtained by picosecond laser writing will also influence the subsequent writing, as shown in the Fig. S2-2e. A large absorbing voxel tends to block part of the light (some angular components of the beam) when another modified spot is tentatively produced at its vicinity. Ultimately, the writing becomes impossible when the processed region is surrounded by already written spots. This can be illustrated by the missing region where the four legs of the Eiffel tower merge. In contrast, these problems are not observed with plasma-seeded approach, which leads to smaller writing voxels using an appropriate femtosecond pre-pulse.

Note 3. Drude model for plasma density estimations

In this note we describe the simple model used to quantify the produced free-carrier densities by local ionization of silicon from the plasma observations.

For simplicity, the observed plasmas are assumed as uniformly ionized regions. Considering the cylindrical symmetry in the studied problem, the thickness (diameter of the cylinder) is directly given by the apparent size of the plasma as observed laterally by infrared microscopy. According to the Drude model, the complex refractive index in the ionized region can be expressed as:

$$\tilde{n}^2 = n_0^2 - \frac{\omega_p^2}{\omega^2 + i\gamma\omega}, \quad (3\text{-}1)$$

where n_0 represents the refractive index of the unexcited material, ω the laser frequency, γ the mean collision frequency, and ω_p the plasma frequency, which can be calculated by:

$$\omega_p^2 = \frac{n_e e^2}{\varepsilon_0 m^*}, \quad (3\text{-}2)$$

where n_e is the free-electron density, e the electron charge, ε_0 the vacuum permittivity, and m^* the effective electron mass. Then, the absorption coefficient can be calculated as:

$$\alpha = 4\pi Im(\tilde{n})/\lambda, \quad (3\text{-}3)$$

where $Im(\cdot)$ denotes the imaginary part, and λ represents the wavelength. Finally, according to the Beer-Lambert law, we can estimate the transmission

$$T = \exp(-\alpha \cdot d_p)\ , \quad (3\text{-}4)$$

where d_p is the thickness of the plasma. In the simulation, we take γ=0.3 fs^{-1}, and $m^* = 0.18 m_e$ (m_e represents the electron mass), which are typical values for low density plasmas in silicon. A summary of the parameters is given in the following table.

Name	Symbol	Value	Literature
Refractive index of the unexcited Silicon	n_0	3.48	[Ref.[3]]
Mean collision frequency [fs^{-1}]	γ	0.3	[Ref.[4]]
Effective electron mass	m^*	$0.18m_e$	[Ref.[5]]

Note 4. Interaction simulations

This note describes the details of the simulation methods and the obtained results for this work.

4.1 Simulation methods

To simulate the highly non-paraxial interactions with high-NA focusing, we adopt the method developed in reference [6]. To simulate the effect of the plasma produced by the first pulse on the propagation of the second one, we use the following approach. First, we simulate the propagation of the first femtosecond pulse focused at 300 µm below the flat Si surface by an objective with NA=0.85. The electric field in time for the first femtosecond pulse is given by

$$E_1(t) = E_0 exp(-\frac{t^2}{2{T_0}^2}) exp(-i\omega_0 t), \tag{4-1}$$

where $T_0 = 190/(2\sqrt{\log 2})$ is the pulse duration (FWHM) taken at 190 fs.

Recombination dynamics is then considered in the double-pulse model. Based on the plasma densities measured by pump-probe shadowgraphy, we fit the time-dependencies by using different recombination models, considering different possible components: exponential decay, two-body recombination, and three-body recombination (equations shown in Fig. S4-1 legend). The results presented in Fig. S4-1. show that the two-body recombination model consistent with an Auger decay gives the best agreement with our measurements. Accordingly, we predict the time-evolution of the plasma density after the first femtosecond pulse by the following equation

$$\rho(t) = \frac{\rho_0}{1+\gamma\rho_0 t}, \tag{4-2}$$

where ρ_0 is the initial plasma distribution obtained in the simulation of femtosecond pulse propagation and $\gamma = 1.7\times10^{-15}$ m^3/s, which is taken from the best fitting conditions for the experimental data. For visualization of the pre-plasma conditions before simulation of the effect of the picosecond pulse, the time *t* in Eq.4-2 is taken as the delay between the femtosecond and chirped pulses (10 ps from peak to peak).

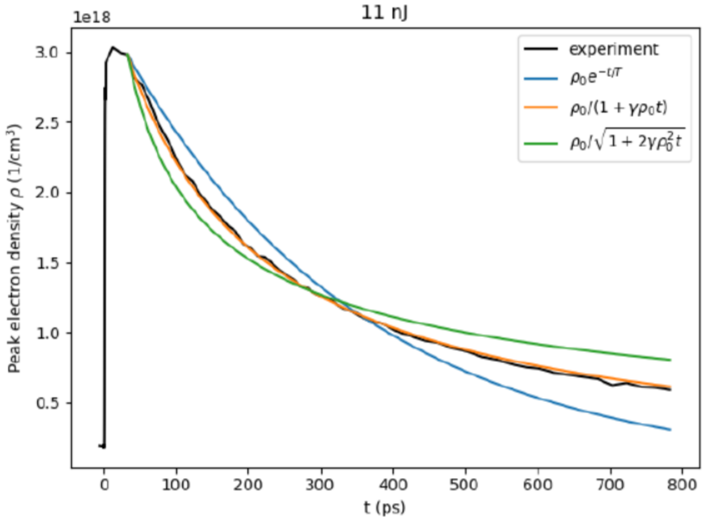

Fig. S4-1. Time-resolved measurement of the free-carrier density compared to different recombination models.

Then, we simulate the evolution of plasma density over time considering the kinetic plasma dynamics. The kinetic plasma equation for electron density ρ is the following

$$\frac{d\rho}{dt} = \sigma_K I^K(\rho_{nt} - \rho) + \frac{\sigma_B}{U_{cr}} * \frac{E^2(\rho_{nt}-\rho)}{\rho_{nt}} * \rho - \gamma * \rho^2 \quad (4\text{-}3)$$

where $\sigma_K I^K$ is the multiphoton ionization rate, ρ_{nt}=5.10^{28} m^{-3} is the atomic density, σ_B is the Bremsstrahlung coefficient and U_{cr}=1.5*U_i where U_i=1.12 eV is the bandgap.

Finally, the interaction of the chirped picosecond pulse is systematically repeated for two cases: without and with the pre-plasma as generated by the femtosecond pulse. Unfortunately, due to 3D simulations requiring large computational resources, it was not possible to perform simulation for the actual 11-ps long second pulse corresponding to the experiments. Instead, the considered second pulse is simply 10 times longer than the first one in the simulations. Despite this important difference, as we will see later on, a relatively good qualitative agreement can be obtained with the experimental observations. This must show simulation conditions where the important mechanisms driving the writing performance are already into play with the simulated shorter pulses.

The electric field in time for the second, longer, chirped pulse is taken as

$$E_2(t) = E_0\frac{T_0}{T_c}\sqrt{1-iC}exp(-\frac{t^2}{2T_c^2})exp(-i\left[\omega_0 t - C\frac{t^2}{2T_c^2}\right]), \quad (4\text{-}4)$$

where $C = \sqrt{T_c^2/T_0^2 - 1}$ is the chirp parameter, and the duration is $T_c = 10T_0$.

Based on the previous method [6], 2D maps corresponding to cross-sections of distributions across the focus are calculated for the delivered fluence, the plasma density, and the delivered energy (absorbed).

The quantity called delivered energy is calculated using the following approach. The work W done by the electromagnetic field forces acting on the charges during time interval dt is given by

$$W = \vec{F}\cdot\vec{dl} = q(\vec{E} + \vec{v}\times\vec{B})\cdot(\vec{v}dt) = q(\vec{E}\cdot\vec{v})dt \quad (4\text{-}5)$$

For continuous charge distribution $q = \rho dV$ with electron density ρ and volume element dV we obtain

$$W = (\rho dV)(\vec{E}\cdot\vec{v})dt = \vec{E}\cdot(\rho\vec{v})dVdt = \vec{E}\cdot\vec{J}dVdt \quad (4\text{-}6)$$

where $\vec{J}=\rho\vec{v}$ is the electric current. Therefore, the work done per unit time, per unit volume, which is to say, the energy delivered by the field to free electrons per unit time, per unit volume is

$$\frac{W}{dVdt} = \vec{E}\cdot\vec{J} \quad (4\text{-}7)$$

Accordingly, we plot the energy delivered per unit volume, as calculated by the equation:

$$\frac{W}{dV} = \int \vec{E}\cdot\vec{J}\,dt \quad (4\text{-}8)$$

4.2 Influence of the pre-plasma on the picosecond pulse interactions

The interaction of the chirped pulse is systematically simulated for two cases: without and with the pre-plasma generated by the femtosecond pulse. Simulations are repeated for three different energies for the chirped pulses: 0.02, 0.04, and 0.08 µJ as shown in Fig. S4-2, 3 and 4, respectively. The choice of the pulse energy is made to describe interactions at peak intensities similar to tested experimental conditions (despite the pulse duration difference). The distributions in Fig. S4-2, 3 and 4 show the resulting the local fluence, plasma density, and delivered energy conditions achieved inside silicon.

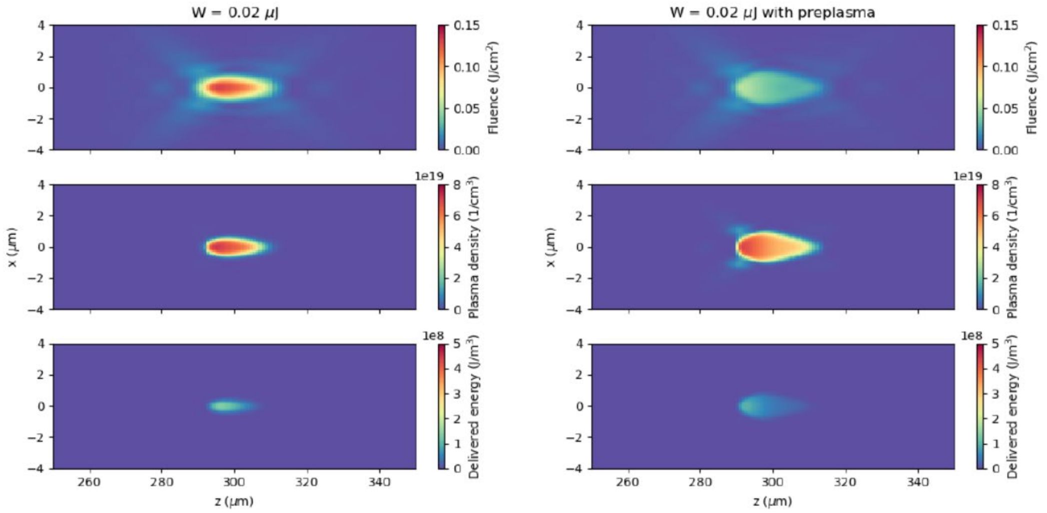

Fig. S4-2. Comparison of the fluence (first row), plasma density (second row) and delivered energy (last row) with a chirped pulse energy of 0.02 μJ.

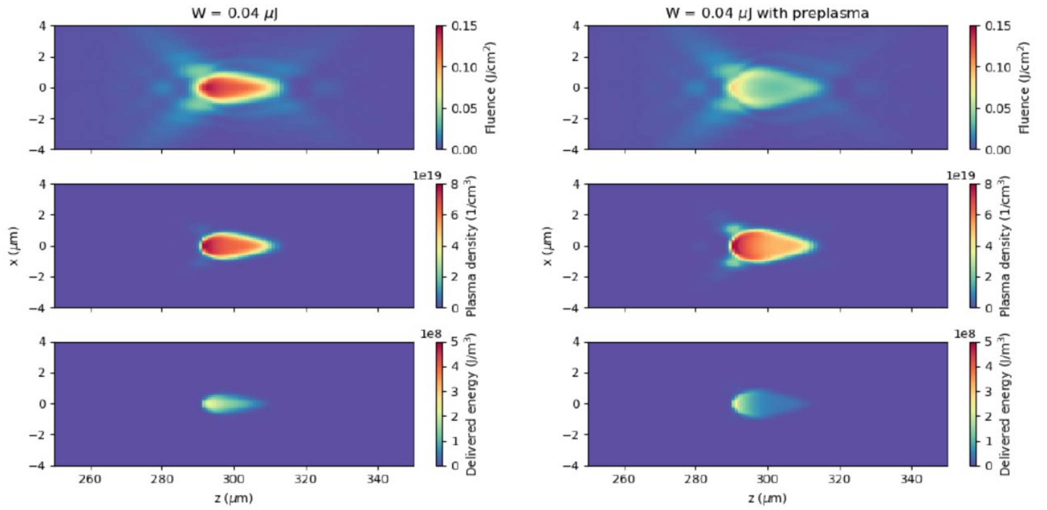

Fig. S4-3. Comparison of the fluence (first row), plasma density (second row) and delivered energy (last row) with a chirped pulse energy of 0.04 μJ.

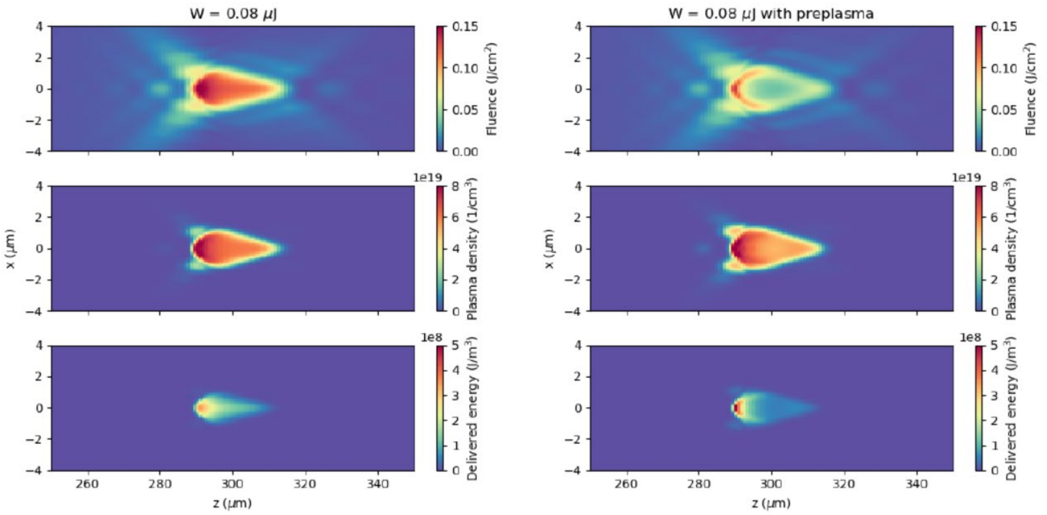

Fig. S4-4. Comparison of the fluence (first row), plasma density (second row) and delivered energy (last row) with a chirped pulse energy of 0.08 μJ.

For better understanding, we plot the 1D dependencies of the peak fluence, peak plasma density, and peak delivered energy along z (the optical axis). Here, we can see that at the low pulse energy of 0.02 μJ, the existence of a pre-plasma plays a destructive role: all peak parameters decrease compared to the case of free propagation. Then, at the energy of 0.04 μJ the peak values for the delivered energy become similar to the free-propagation case even if some spatial distortions are already observed. Finally, at the highest tested energy of 0.08 μJ we observe the benefits from plasma seeding. The peak delivered energy in case of the chirped pulse in presence of the pre-plasma overcomes the corresponding value for the free-propagating pulse. Additionally, we see that the delivered energy forms a better localized peak which explains the decrease of the modification volume obtained in the experiments. The localization of the delivered energy is well seen in Fig. S4-4. In addition, it is interesting to note with these analyses that the peak fluence for the chirped pulses with pre-plasma is always smaller than without pre-plasma. This means that the absorbed energy resulting from electron acceleration in the plasma by the chirped pulses (joule heating) plays an essential role in the local and efficient modification process.

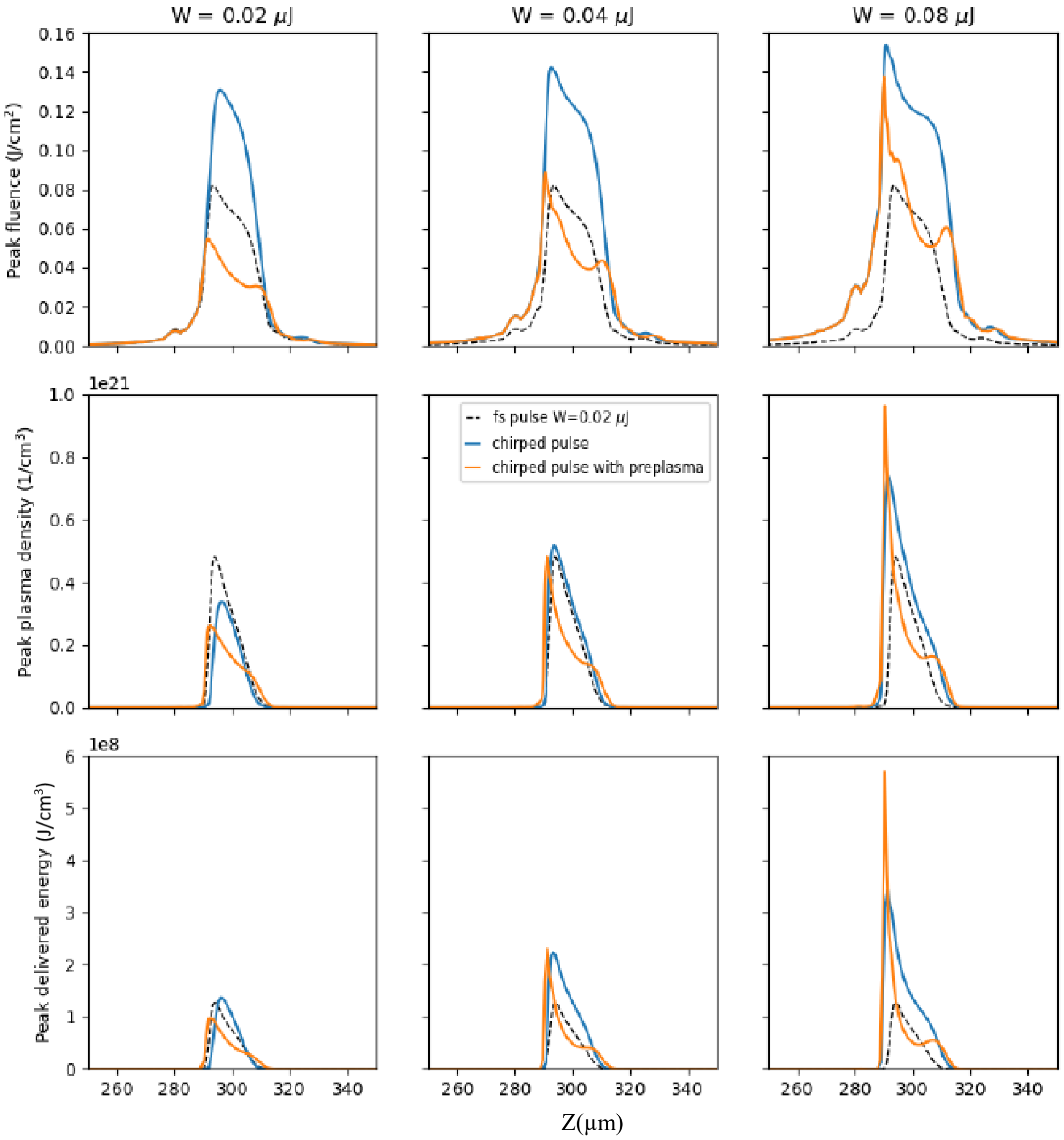

Fig. S4-5. On-axis distribution of the fluence (first row), plasma density (second row) and delivered energy (last row) with different chirped pulse energy.

4.3 Potential propagation effects resulting from plasma seeding

More than pure absorption seeding effects, we can expect that refraction or phase changes resulting from pre-plasma formation in the pre-focal region can reshape the energy flux leading to improved interaction localization. To understand how the pre-plasma conditions influence the energy flux of the chirped pulse, we simply calculate the difference between the fluence distributions obtained with and without the plasma, as shown in the Fig. S4-6. The result shows that the energy flux is not affected by pre-ionization until the plasma front is reached. This unambiguously excludes the possibility that the improved precision obtained by plasma seeding originates from propagation effects improving the focusing conditions.

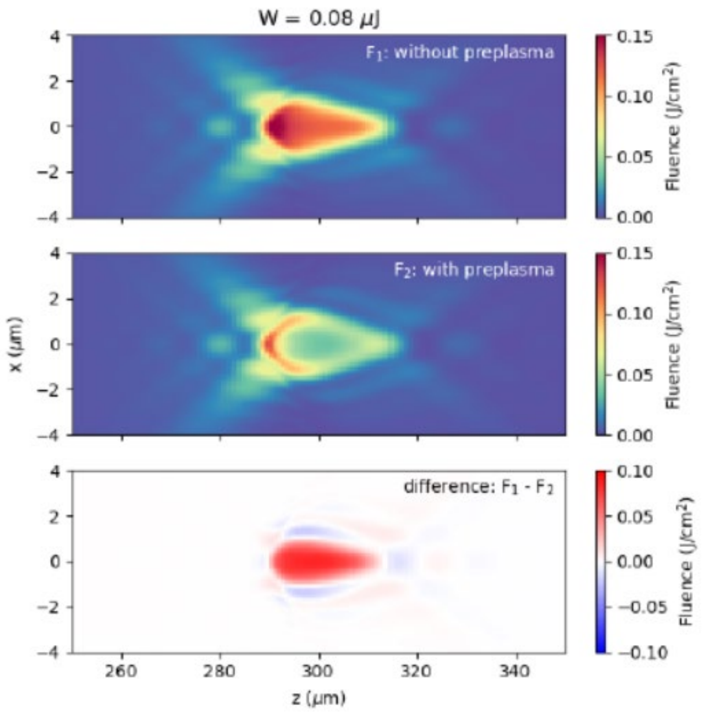

Fig. S4-6. Difference between the fluence maps obtained without and with pre-plasma seeding. The result indicates negligible changes away from the focal zone.

4.4 Simulated modification positions for different plasma sizes

In the experiment, the increase of the energy of the femtosecond pulses results in a shift of the modified spot in the backward direction and, at high enough energy, to zero damage probability (see the Fig. 2e in the manuscript).

To understand this phenomenon, we simulate the interactions of picosecond pulses with an energy of 0.08 μJ and delayed by 10 ps with respect to pre-plasmas generated by femtosecond pulses with different energies. Fig. S4-7 and S4-8 show that the increase of the femtosecond pulse energy causes a decrease of the peak delivered energy. Also, there is a shift on the location of the peak of the delivered energy but it remains modest and very difficult to directly observe on these 2D colour maps. To quantitatively show the effective shift, we plot the on-axis delivered energies as function of the femtosecond pre-pulse energy in Fig. S4-9a. We can see that the peak of the delivered energy shifts away from the focal spot, towards the air-silicon interface. The graph in Fig. S4-9b shows the absolute values of the shifts as function of femtosecond pulse energy, relative to the case without plasma seeding. All the conclusions from these simulation analyses are consistent with the experimental observations.

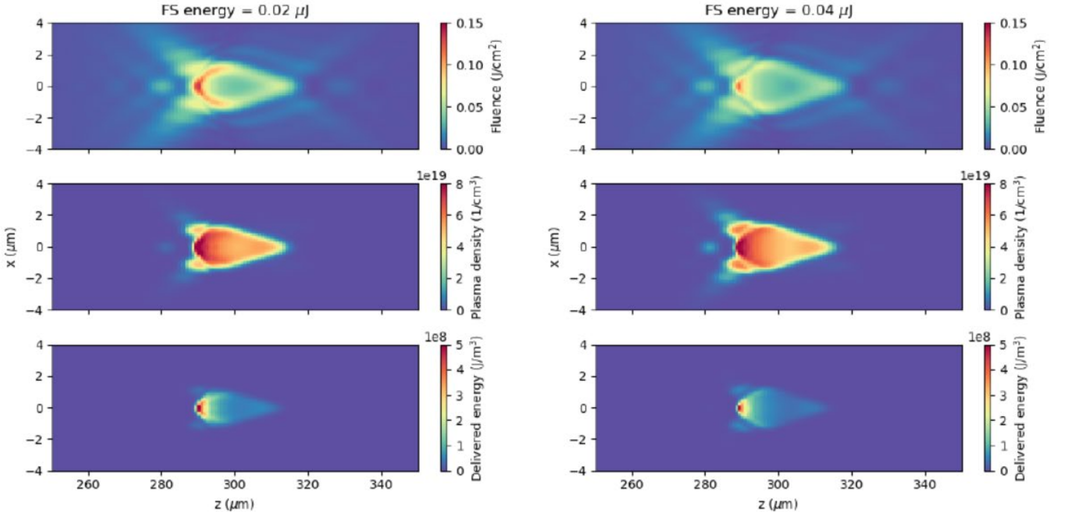

Fig. S4-7. Comparison of the fluence (first row), plasma density (second row) and delivered energy (last row) with a femtosecond pre-pulse energy of 0.02 µJ (left) and 0.04 µJ (right).

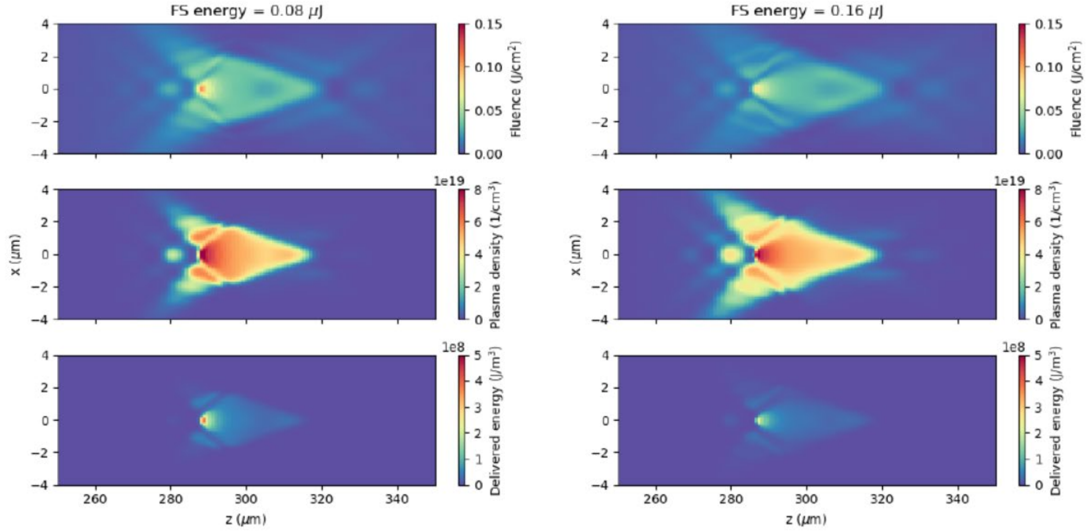

Fig. S4-8. Comparison of the fluence (first row), plasma density (second row) and delivered energy (last row) a femtosecond pre-pulse energy of 0.08 µJ (left) and 0.16 µJ (right).

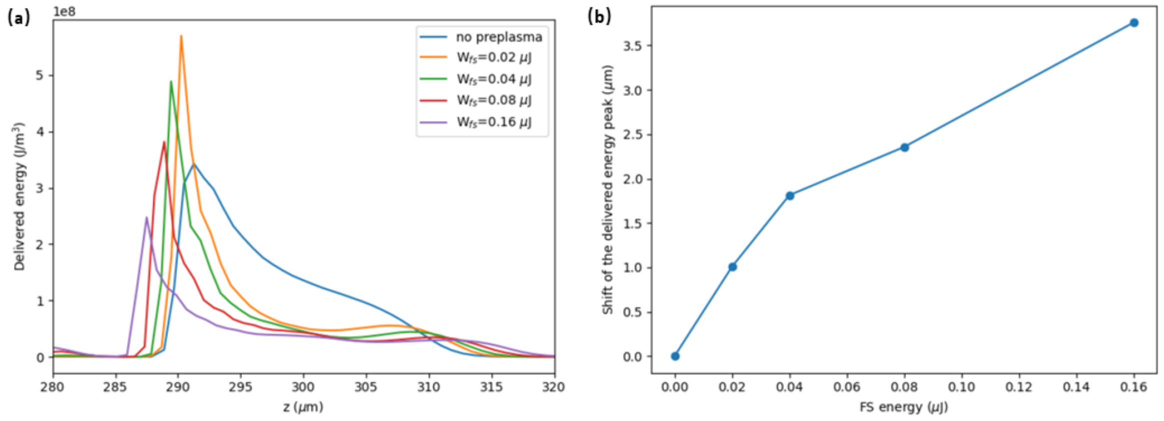

Fig. S4-9. On-axis distributions of the delivered energy with different femtosecond pulse energy (a) and their peak positions (b).

Note 5: Methods for relative spot motions

This note describes the methods employed for relative change of the positions of the femtosecond and picosecond spots away from perfect focus overlap. The temporal delay is measured by autocorrelation to ensure the same temporal delay in these experiments.

5.1 Lateral shift (in X)

As shown in Fig. S5-1 the relative beam pointing directions are controlled by tilting the beam splitter recombining the beams and placed very close to the focusing objective lens (< 3 cm). Depending on the experiments, the beam paths for the femtosecond and picosecond pulses are switched to preferentially move one beam with respect to the other.

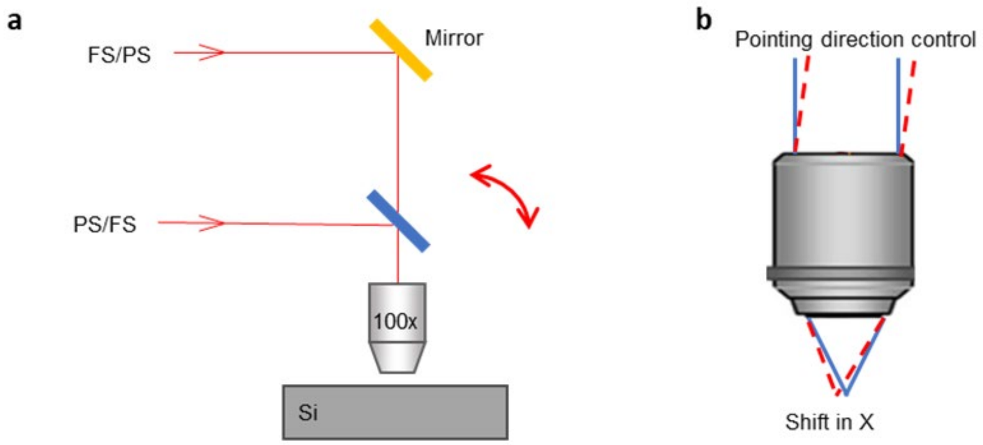

Fig. S5-1. Spatial control of the plasma position in X. a. The beam pointing direction controlled by tilting the beam splitter (marked by the red arrow). **b.** An example of the beam shift in X direction.

5.2 Longitudinal shift (in Z)

In this work, different plasma positions along the optical axis are also tested. This is done by changing the divergence of the femtosecond beam as shown in Fig. S5-2. The divergence is precisely controlled by inserting a telescope consisting of two identical bi-convex lenses and changing their relative distance. Then the focal position can be estimated by simple ray-tracing methods.

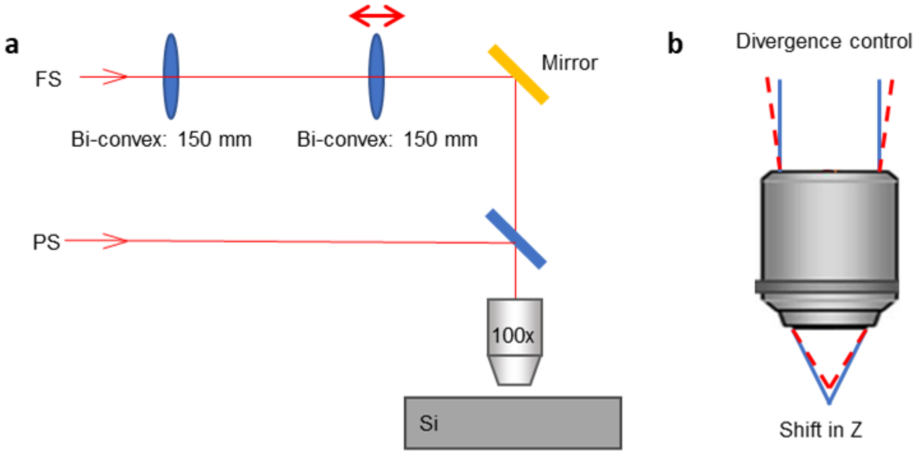

Fig. S5-2. Spatial control of the plasma position in Z. a. The divergence control by shifting one of the lenses (marked by the red arrow) in the telescope. **b.** An example of the beam shift in Z direction.

Note 6. Characterizations of modifications

This note describes the methods and details of the performed material analyses.

6.1 Raman Spectroscopy

Sample preparation methods and Raman measurements. The excitation wavelength of Raman micro-spectroscopy systems usually lies in the visible range which is not appropriate for in-bulk inspection of silicon. Therefore, a necessary step to analyse the structures inside the bulk is to expose the bulk structures to the surface. For this purpose, we apply scanning approaches to write large modified volumes in the bulk and then cleave the wafer along a plane intercepting the structures. A cross-section of some structures can then be exposed at the surface as can be seen in Fig. S6-1a.

The structures are then characterized by Raman micro-spectroscopy with an excitation wavelength of 532 nm. The images are obtained in a scanning mode with a step size around 5 μm and an accumulation time of 0.1 s. Fig. S6-1 shows some of the obtained results.

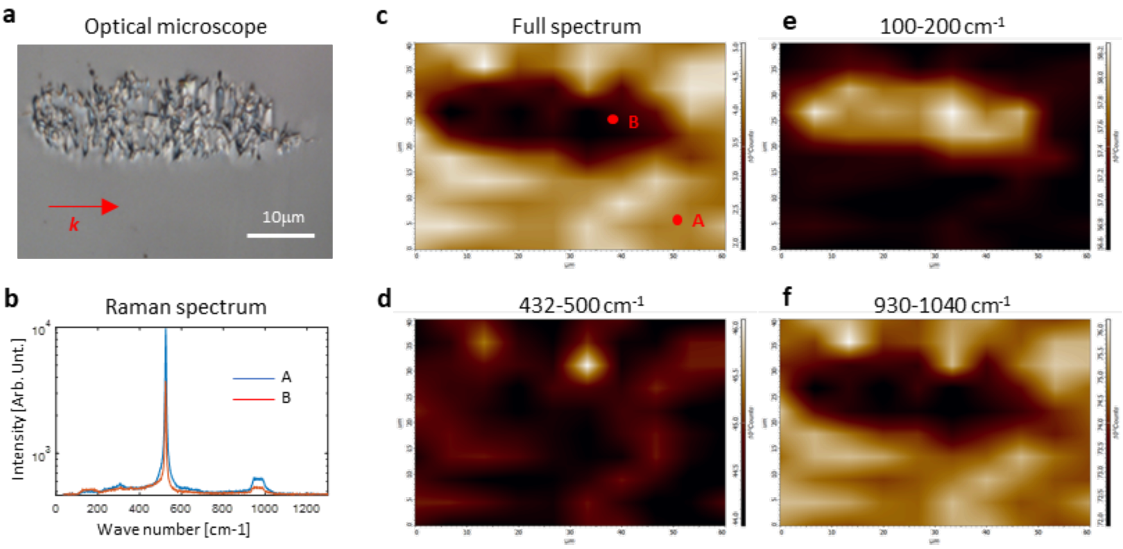

Fig. S6-1. Measurement of Raman spectrum. **a**. Optical microscope image of the cross section. **b**. Spectrum comparison between pristine Si (A) and the modified area (B). **c**. An accumulated spectrum signal ranging from 50 cm^{-1} to 1400 cm^{-1}. **d-f**. Accumulated spectrum at specified range.

Comparison between modified and pristine area. Specifically, important features of crystalline structure of silicon are observed with the plateau region from 930-1040 cm^{-1} whereas the region between 100-200 cm^{-1} is related to the amorphous phase. More characteristics from the Raman peaks of silicon can be discussed using reference [7]. However, the opposite contrasts obtained between Fig. S6-1e and f can be directly interpreted by an amorphization process of Si (even if partial) with laser writing.

6.2 Transmission electron microscopy (TEM)

As can be seen in Fig. S6-2, a thin lamella of material containing the modifications was obtained with Gallium focused ion beam (FIB), perpendicularly to the edge of the sample after a platinum protective layer deposition (electronic and ionic). Then, TEM images were realized in bright field (BF) at 300 kV with a contrast diaphragm (50 μm), after orientation of the lamella according to monocrystalline silicon.

The structure was studied with FFT of TEM images and also in diffraction mode. The TEM observation is shown in Fig. S6-3, with the reference area on the left and the laser-modified region on the right of the image. Specific parts of the lamella marked by red squares are then magnified for presentation in Fig. S6-4a. Electron diffraction is then conducted, confirming the monocrystalline nature of silicon in the reference region (Fig. S6-4a). A very small part of amorphous silicon is also detected (see FFT on Fig. S6-4c), maybe induced by FIB preparation or scattered laser writing pulses.

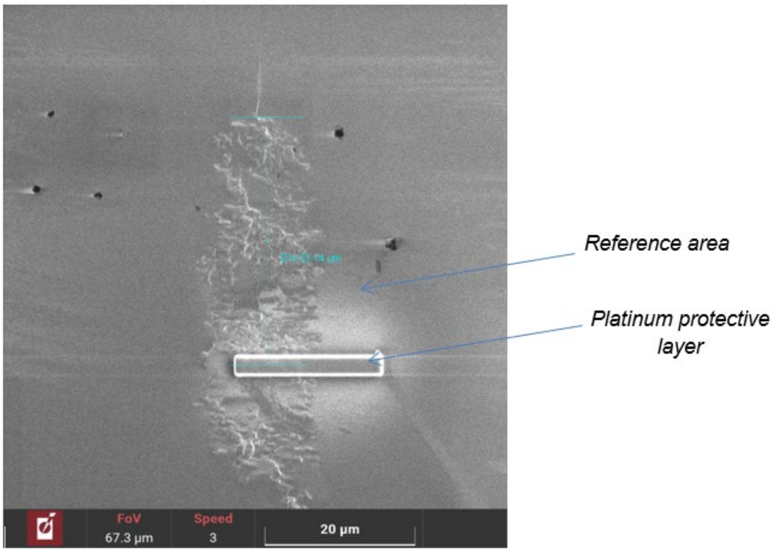

Fig. S6-2. FIB image after platinum protective layer deposition of the sample.

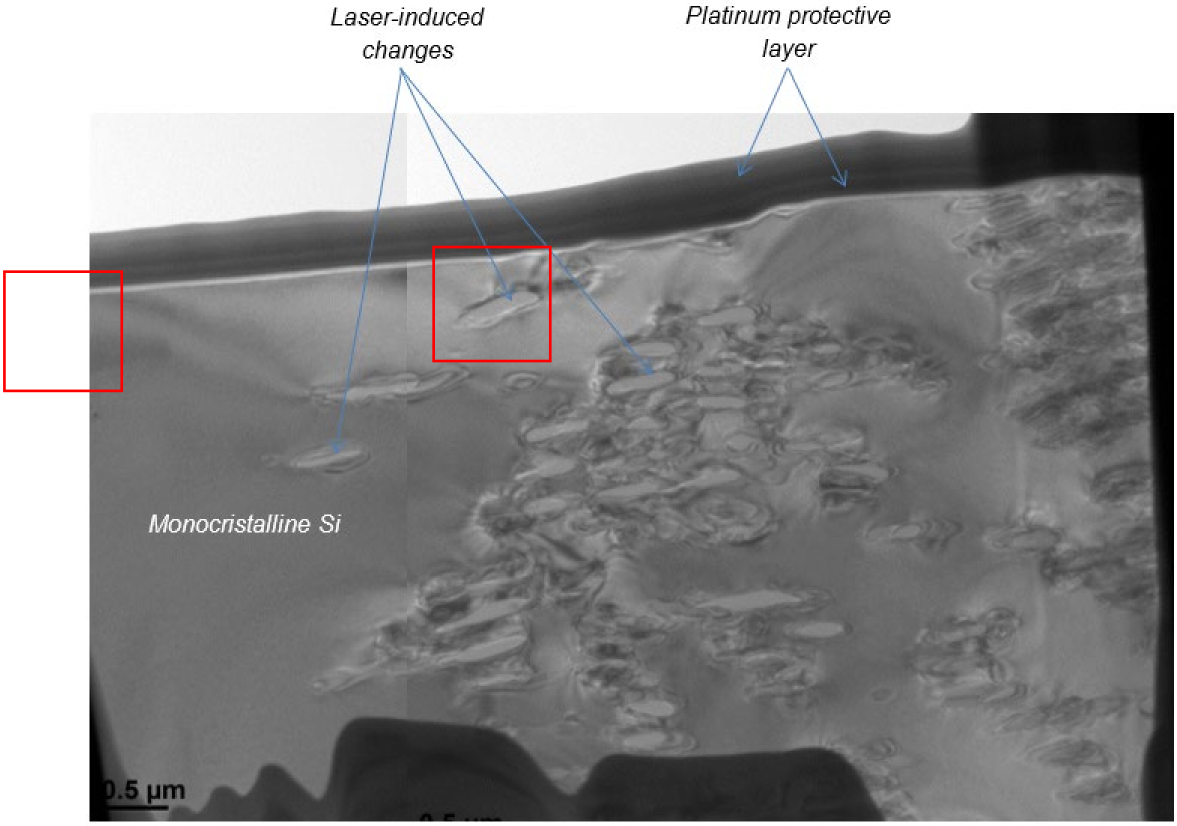

Fig. S6-3. TEM-Bright Field (300 kV) of the lamella. The two red boxes are magnified in the following figure.

For the zone of the lamella corresponding to laser modified region the presence of several oval defects appearing as bright regions surrounded by darker non-uniform affected zones is immediately noticed. The chosen magnified zone reveals the details of a typical defect (right red box in Fig. S6-3 corresponding to Fig. S6-4b) for further investigations. Fig. S6-4b shows that the defect is 560 nm long and 130 nm large. Two areas were analysed by electron diffraction: Area 2 is just outside of the defect, showing that it is still crystalline but exhibiting a larger amount of amorphous silicon compared to area number 1 corresponding to the reference (see FFT on Fig. S6-4d). Area 3 is taken directly inside the defect, showing a completely amorphous structure (see FFT on Fig. S6-4e). Moreover, an amorphous layer (~ 45 nm thick) is visible on the surface of the lamella, between crystalline silicon and the platinum protective layer (images 4a and 4b). However, this layer is observed for the reference and modified zones and then must correspond to a layer of silicon oxide.

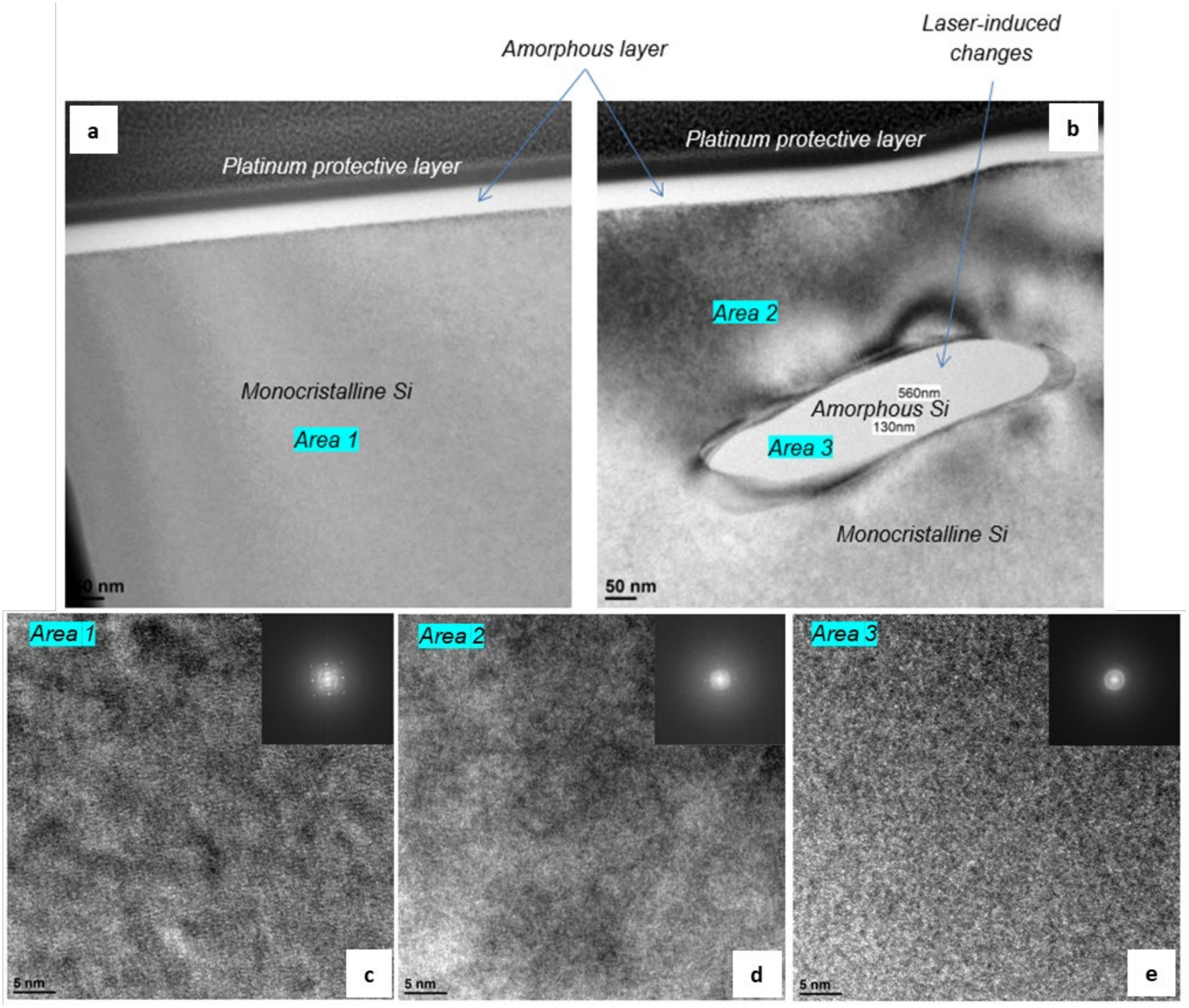

Fig. S6-4. Magnified view of the structures and their electron diffraction test. a-b. Magnification of the red boxes in the previous figure. **c-e**. Magnified TEM results marked in a-b, with their electron diffraction test displayed at the upper right corner.

An interesting observation about the amorphous domains is their direction and periodicity. A first obvious observation is that all the amorphous grains elongate along the same direction. Additionally, we find that it is possible to note an apparent periodicity along lines in the assembly of produced amorphous grains. To quantitatively reveal these features, we apply an image processing method on the image shown in Fig. S6-5a to conserve only the amorphous grains in Fig. S6-5b. The superimposed periodic lines to guide the eyes already confirm the periodicity. To study this aspect, we also conduct

2D fast Fourier transformation of the image leading to the results shown in Fig. S6-5c. The central part of the spectrum is then magnified and the contrast adjusted to obtain Fig. S6-5d. This unambiguously reveals two peaks, indicating the periodicity of 255 nm and a normal direction along 100 degrees. These characteristics match very well with the lines presented in Fig. S6-5b.

Comparing with the polarization direction of the writing pulses, we find that the amorphous grains elongate along the polarization direction. This relationship is consistent with a previous work reporting secondary scattered electron microscopy results on modifications induced by modest-contrast strong femtosecond laser pulses. These previous works reveal also periodical small change of silicon properties with similar orientations[8].

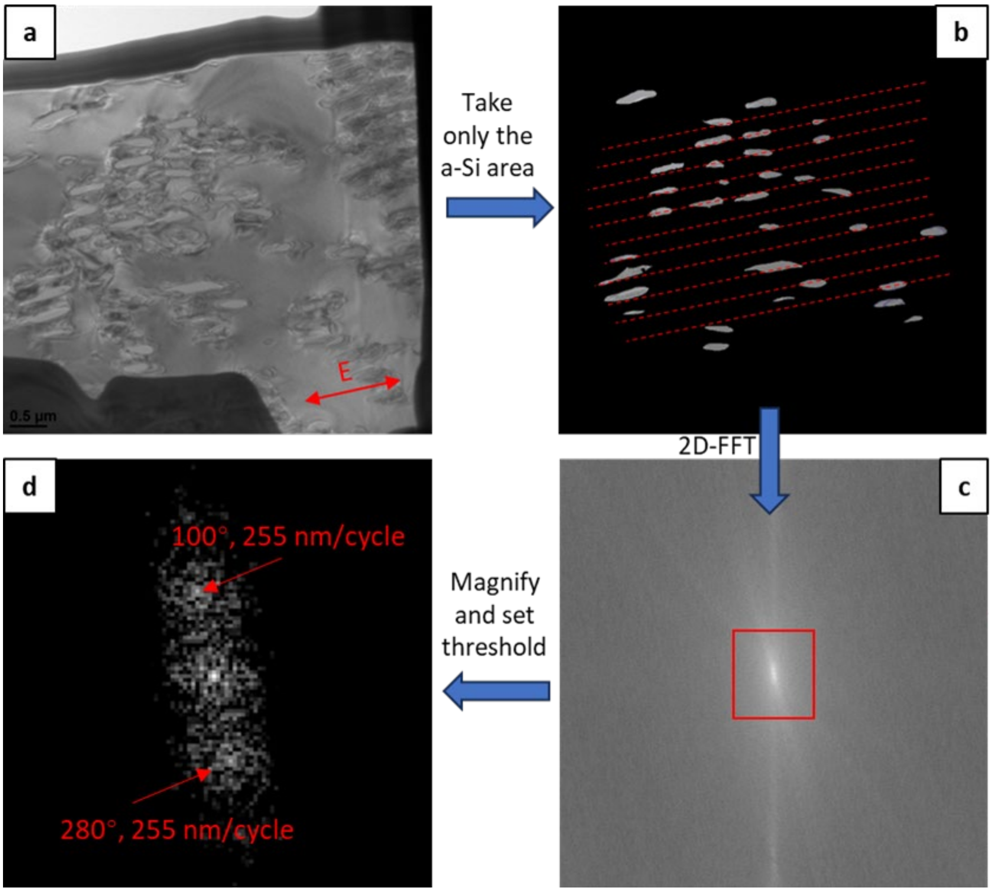

Fig. S6-5. Periodicity and direction of the laser written amorphous structures. **a.** TEM results revealing the formation of aligned grains of amorphous Si. The polarization of the writing pulses is shown at the bottom right. **b.** Image conserving only the amorphous grains. **c-d.** Results of FFT applied on image b and their magnification. The grey scale is arbitrarily adjusted to improve the image contrast in **d**.

Note 7. Laser erasing of modifications

This note describes the experimental considerations for erasing the produced modifications using nanosecond laser irradiation.

7.1 Setup and Method

Experimental arrangement. In the setup, we use the femtosecond and picosecond double-pulse sequence at 1550 nm for writing modifications inside Si. As shown in Fig. S7-1a, a combination of half-wave plate (HWP) and polarizing beam splitter (PBS) is used to change the writing pulse energy. For erasing, we add a nanosecond laser (MWTech, PFL-1550, pulse duration 5 ns, 1550 nm) using a beam splitter as an alternative source. As shown in the main text of the paper and later in this note, the thermal nature of interaction in this long pulse regime permits the erasure of the structures written with the double-pulse configuration. In practice, automatically-actuated mechanical shutters in the beam paths (see beam dumpers BD in Fig. S7-1) are used for independent control of the writing and erasing processes during the experiments.

The writing and erasing processes can be precisely overlapped as both spots are produced by the same focusing optical elements. The spatial overlap is checked by methods mentioned in Supplementary Note 1. After irradiation, the modifications are inspected by an infrared microscopy system (InGaAs camera) using bright field (BF) and dark field (DF) modes of observation. A typical image of the produced modifications is shown in Fig. S7-1b. Irradiations are repeated to form a 5x5 matrix of identical modifications. In this case the energies of the applied femtosecond and picosecond pulses are respectively 0.18 µJ and 0.35 µJ and the temporal delay between pulses is 20 ps (femtosecond pulse is first). In Fig. S7-1c the same matrix is presented after tentative erasure of each spot by applying 1000 pulses of 0.2 µJ. The DF image supports the erasing process largely supressing light scattering from the modified zones.

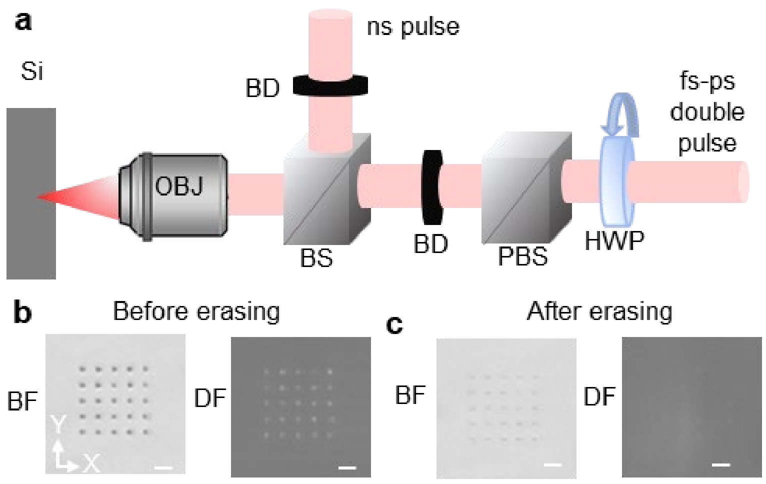

Fig. S7-1. Simplified representation of the configuration for writing and erasing micro-modifications inside silicon. a. Experimental arrangement to precisely overlap writing and erasing beams. **b.** Bright field (BF) and dark field (DF) infrared microscopy images of a matrix of produced modifications. **c.** Same image after applying on each spot erasing nanosecond pulses. The scale bar is in 10 µm.

Evaluation of the erasing effect. The parameters for erasing with the nanosecond pulses are investigated. In particular, the number of applied pulses, the pulse energy, and spot position strongly influence the erasing efficiency. For the optimization studies, we needed to define a parameter to quantitatively evaluate the erasing efficiency. We introduce the so-called *erasing potential* parameter according to the equation,

$$Erasing\ Potential\ (\%) = \left[1 - \frac{(P_{er} - P_{ref})}{(P_{wrt} - P_{ref})}\right] \times 100, \qquad (7\text{-}1)$$

where P_{ref}, P_{wrt}, P_{er} are the scattering signal values corresponding to the modified zones (measured pixel signal in DF) before and after writing and after erasing. By systematically evaluating this quantity we optimized the different control parameters of the erasing beam as described in the next sections.

7.2 Laser conditions for efficient erasing

7.2.1 Number of applied nanosecond pulses

To illustrate the effect of the number of applied laser pulses for the erasing process, we first show in Fig. S7-2a a bright field image of a 3x10 matrix of individual modifications all written with the same double-pulse sequence (as explained in previous section). The corresponding DF image, defines the scattering signal associated with modified spots. For the erasing process, the number of applied nanosecond pulses is then taken as a variable parameter. It varies linearly from 10 to 100 with a step size of 10 for the different columns presented in Fig. S7-2b. From the BF and DF image, we can see that the erasing process continuously improves with the number of applied pulses in this range. Interestingly, one can notice from the observations that the minimum pulse number to initiate significant erasing is about 30 pulses.

For quantitative evaluation of the erasing capability we rely on the scattering signal responses in the DF images and use Eq. 7-1. From, the images presented in Fig. S7-2, we can extract the maximum scattering signal through the maximum pixel value in each modified zone. As an illustration we present the extracted raw values from Fig. S7-2a in Fig. S7-3.

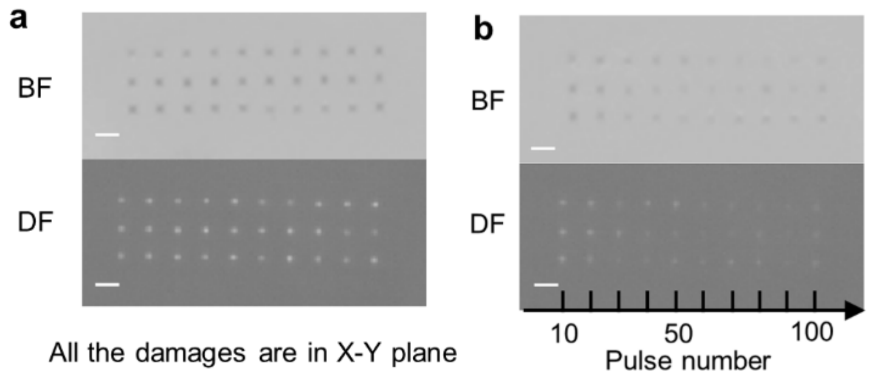

Fig. S7-2. Pulse number dependent erasing results. a. BF and DF microscopy images of a 3x10 matrix of identical modifications produced inside Si. **b.** Similar images after tentative erasure varying the number of applied nanosecond laser pulses. All the scale bars are 10 µm.

First one can note relatively important fluctuations between measured scattering signals despite identical writing conditions. However, after averaging on 3 sites, a relatively clear trend can be derived as can be

seen in Fig. S7-4a presenting the measured scattering levels before and after erasing. In particular, we can see the gradual decrease of average scattering signal with the increase of the number of applied pulses for writing (marked by the red histogram distribution). Interestingly, the reference line in Fig. S7-4a corresponds to the average scattering level for an unmodified scattering region (20x20-pixel area) in the same DF image and referred as P_{ref}. From Eq. 7-1, the erasing potential is then calculated based on this and other measured parameters. In Fig. S7-4b, the erasing potential can then be plotted as function of the number of applied pulses leading to the conclusion of an optimum is reached for about 40 pulses. The erasing potential remains in the range of 75-88 % for all tested conditions above this number.

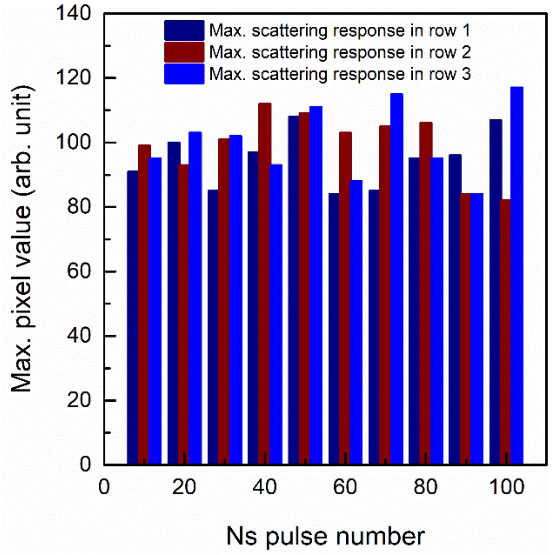

Fig. S7-3. Scattering signal from modifications after writing. General maximum scattering response (in pixel signal values) for modifications written in a 3x10 matrix all with similar conditions. Data are extracted from the DF image presented in Fig. S7-2a.

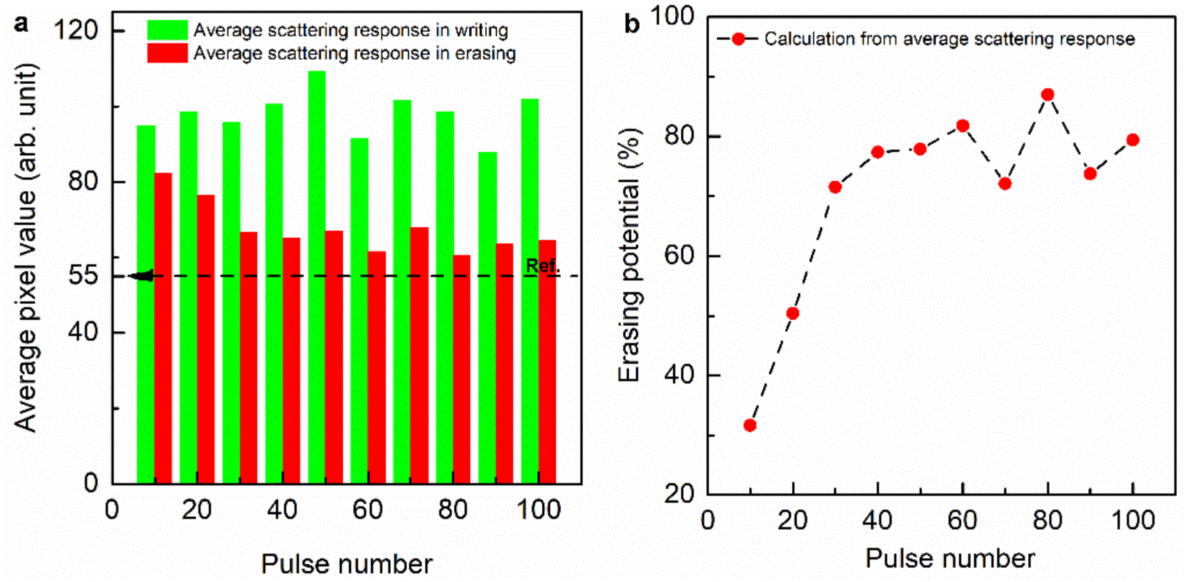

Fig. S7-4. Erasing efficiency as function of number of applied nanosecond laser pulses. a. Average scattering signal from modification after writing and erasing as determined by DF microscopy imaging. **b.** Evaluation of the erasing potential according to Eq. 7-1 as a function of the number of erasing pulses.

7.2.2 Nanosecond pulse energy

Obviously, the erasing also depends on the applied nanosecond pulse energy. The importance of this parameter can be directly seen with the modifications shown in Fig. S7-5a. In this case, the maximum pulse energy at 100 % level corresponds to 0.5 µJ, which is about half of the writing threshold (1.26 µJ) inside Si with the 0.85 NA objective using the same laser. From the tested pulse energies from 0 to 100% with 10 % steps we can note that the erasing results are first improved. Then, above 70% energy level, the erasing effect degrades. To quantitatively confirm these observations, we have evaluated for each tested conditions the erasing potential. The results are presented in Fig. S7-5b showing a maximum at about 83 % for a pulse energy of 0.2 µJ (40 %). Above this pulse energy, the erasing potential diminishes progressively, because of conditions leading to the growth of pre-existing modifications more than their erasing.

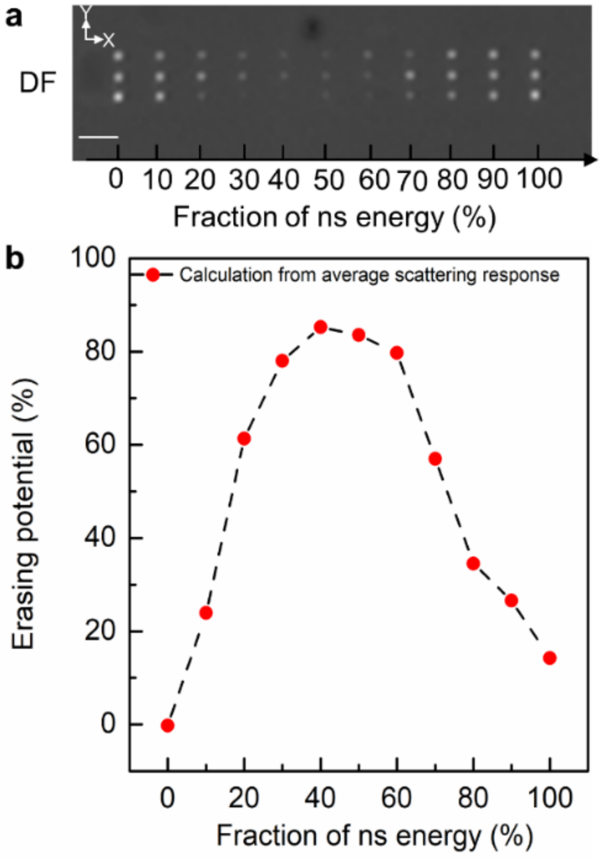

Fig. S7-5. Erasing as a function of nanosecond pulse energy. a. DF microscopy image of modifications after tentative erasing of modifications with different nanosecond pulse energy levels. The scale bar is 10 µm. **b.** Erasing potential as function of pulse energy level as calculated from the above DF image.

7.2.3 Nanosecond focus positioning

Given the small focal spots used in these experiments, the precision in the spatial focusing position strongly influences the erasing process. At first, the depth of focusing of the erasing laser beam (along Z axis) is considered for optimizations. As shown in Fig. S7-6a variation of the focusing depth of few micrometres can strongly influence erasing efficiency. In this case, the position Z=0 corresponds to the erasing beam when focused exactly at the same position as the writing beam (geometrical focus).

However, it is striking to note the low erasing capability with this theoretically perfect overlap. It is only when the nanosecond beam is irradiated deeper (1-µm steps along Z axis shown in Fig. S7-6) that we can get better conditions for erasing. An optimum is found at a depth of ~5 µm in this case. From Fig. S7-6b presenting the corresponding erasing potential as function of focusing depth position, we can see that if we go deeper than 5 µm the scattering response gets larger, suggesting a decreasing of the erasing performance but also potentially the growth of modifications with the applied nanosecond pulses. Taken together, these observations illustrate the importance to consider the focusing depth for optimum local erasing conditions. Similarly, we investigated the required precision on beam position in the XY plane (lateral) and conclude that the erasing effect vanishes in the studied cases if the focus position is only ~1 µm away from the best overlap.

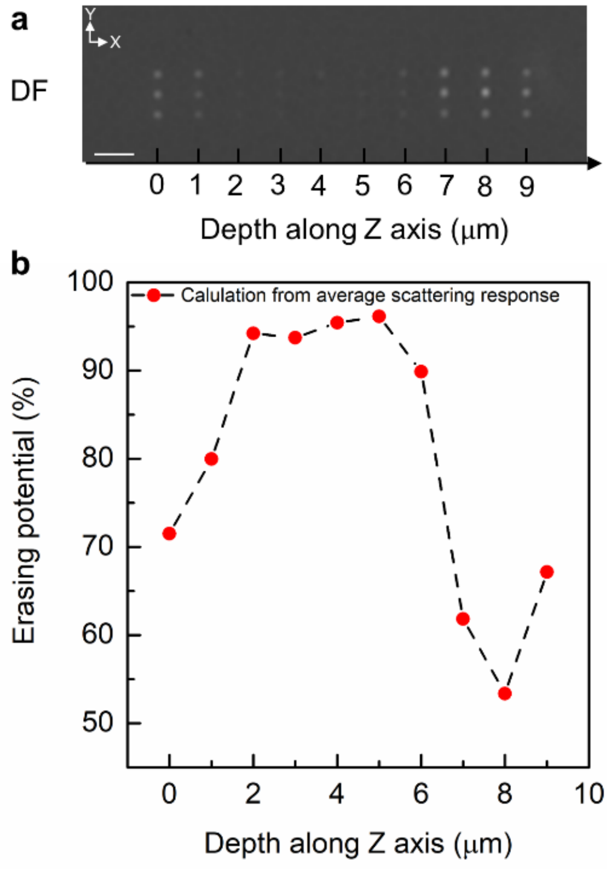

Fig. S7-6. Erasing as function of focusing depth. a. DF microscopy image of modifications after tentative erasing with different focusing depths for the nanosecond laser. Z=0 corresponds to the nanosecond laser focused at the same depth as the writing beam. Scale bar is 10 µm. **b.** Erasing potential as function of focusing depth for erasing as calculated from the above DF image.

7.3 Type of written features

The erasing capability is also a function of the type of the produced modifications depending on the writing conditions. To study this aspect, we have produced modifications using the double-pulse writing configuration and varied the number of applied pulses for writing. This is shown with the 3x10 matrix in the dark field image presented in Fig. S7-7a. Generally, if we increase gradually the number of pulses from 10 to 100 (linear increase from one column to each other), we can see an increase of scattering from the modified region attributed to an increase of the defect density and increasing modified volume. When tentatively erasing the structures using the best conditions for the nanosecond laser (described before, pulse energy of 0.2 µJ, 1000 pulses in 1 second exposure time), we note with the comparison

between DF images in Fig. S7-7a and b that it is possible to erase the modifications produced with 10 pulses. However, stronger modifications obtained with more pulses progressively become more difficult to erase. For quantitative analysis, we again compare the measured erasing potential. As shown in Fig. S7-7b, the erasing potential remains above 65% for writing conditions up to 90 applied pulses before a sharp drop down is observed. In addition, we have noticed the possible occurrence, when we erase, of small defect formation in the vicinity of previously written spots with more than 50 pulses when we erase. This leads to an erasing capability less robust and less stable in addition to the decreased efficiency for modifications produced with higher number of pulses.

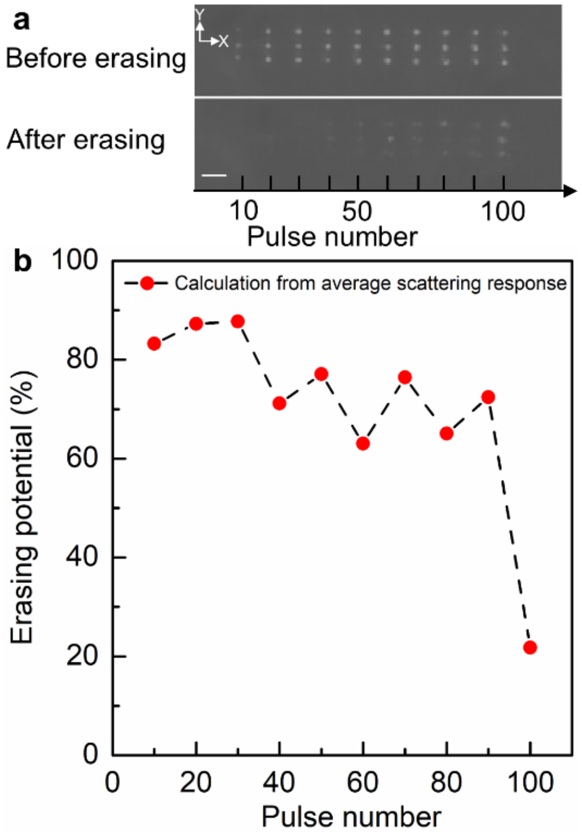

Fig.S7-7. Erasing as function of the number of applied pulses for writing. a. DF microscopy images after writing using different number of double pulses and after erasing using fixed nanosecond laser conditions. Scale bar is 10 µm. **b.** Erasing potential as function of number of pulses for writing as calculated from the above DF image.

7.3 Inscription of QR codes inside Si

After showing the capability to achieve writing/erasure cycles inside silicon, we have exploited this feature for the demonstration of rewritable QR codes. The number of applied ultrafast double-pulses for writing is 10 and we use the pre-optimized nanosecond laser conditions (described before) for erasing (50% energy, 1000 pulses, 5-µm focus shift). For this demonstration, we have adopted a procedure where we systematically write and erase only the needed pixels to create the desired new QR code. This is illustrated in Fig. S7-8a through a simulation format (processing plan). In Fig. S7-8b we can observe the first laser-written QR code which can be read using any standard QR code scanner to reach the LP3 website. In the next BF image, we have written the spots which do not overlap with the previous QR code points so that in the next step after an erasing sequence we can directly get the next QR code reaching the Aix-Marseille university website. In Fig. S7-9, we show the images in BF and DF after successive inscriptions of 3 QR codes at the same location inside the silicon substrate.

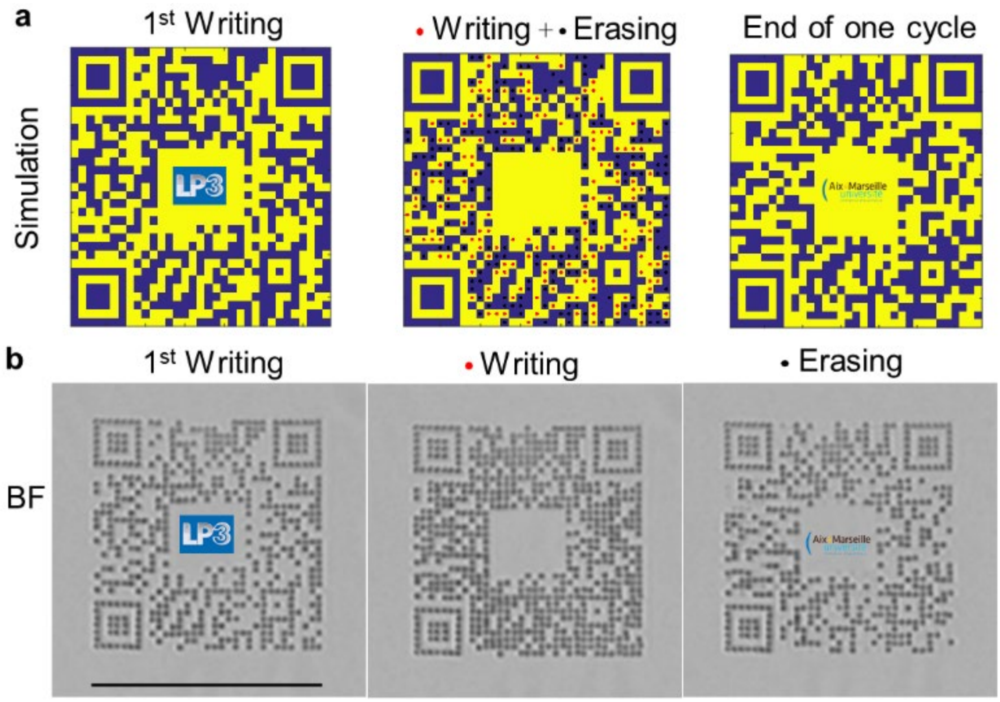

Fig. S7-8. Laser writing of reconfigurable QR codes inside Si a. Simulated QR code points in grayscale format where writing and erasing sequences are described to change the information from the LP3 to the Aix-Marseille University URL. **b.** Corresponding BF infrared microscopy images of the laser written QR codes inside Si. The scale bar is 100 µm.

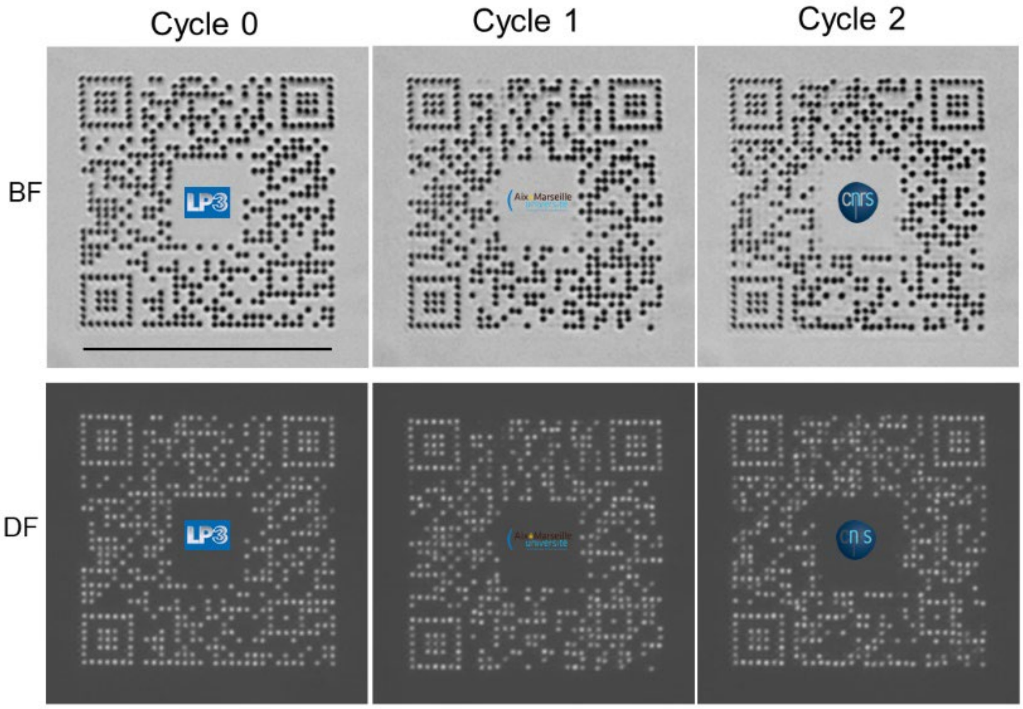

Fig.S7-9. BF and DF infrared microscopy images of inscribed QR codes for LP3, Aix-Marseille University and CNRS URLs at the same location inside a silicon substrate by applying optimized laser writing and erasing sequences. The scale bar is 100 µm

Note 8. Phase-plate fabrication, measurement and applications

In this note we describe the experimental details for quantitative phase measurements and the phase plate fabricated using the double-pulse laser writing strategy.

8.1 Methods for quantitative phase infrared imaging

In the experiments, we have fabricated phase plates inside silicon using the proposed double-pulse strategy and then compared them to similar realisations applying conventional single-pulse laser writing. The plates are characterised by a specially developed infrared interferometric microscope to map in two dimension the phase delay induced by laser writing[9]. The setup is shown in Fig. S8-1. Light from an infrared coherent light source (wavelength of 1317 nm, Thorlabs SLD) is split by a polarizing beam splitter (PBS) into two beams. One of the beams propagates through a quarter-wave plate (QWP) and is reflected by a gold mirror (M1) mounted on a nanometre-precision piezoelectric stage. With two passes through the quarter-wave plate, its polarization direction is rotated by 90 degrees. Accordingly, the reflected light is transmitted through the PBS leading to two separated beams in both arms of the interferometer and exhibiting the same polarization. After passing through identical micro-objective lenses (MO) and being recombined by the beam splitter (BS), the two beams interfere on the InGaAs camera (Raptor, OWL SWIR 640, pixel size of 15 μm). The intensity distribution $I(x,y)$ forming the image is modulated accordingly to the interference response given by:

$$I(x,y) = A_o^2(x,y) + A_r^2(x,y) + 2A_o(x,y)A_r(x,y)\cos[\Delta\varphi(x,y) + \varphi_{pz}] \quad (8\text{-}1)$$

where $A_o(x,y)$ and $A_r(x,y)$ correspond to the amplitude distribution of the object and reference beams, $\Delta\varphi(x,y)$ corresponds to the phase difference between the object and reference, and φ_{pz} is the uniform phase delay introduced by the movement of the piezo stage. To retrieve the phase image, we then acquire four interference images by precisely increasing by π/2 the phase delay using the piezo stage. Then the phase map can be calculated as:

$$\Delta\varphi = \arctan(I_4 - I_2)/(I_3 - I_1) \quad (8\text{-}2)$$

where I_k (k=1,2,3,4) is the measured intensity map of when $\varphi_{pz} = (k-1)\pi/2$. Theoretically, we can directly retrieve the phase image form this procedure. However, in practice, there are some other sources of phase differences that originate from the imaging system. To get rid of them, we first measure a background with a reference sample and then subtract the background from the phase map of the objective sample. One example of the phase retrieval is shown in Fig.S8-1b. The results show a high-resolution phase measurement of two stripes fabricated by modification at different pulse energies. It is worth noting some phase jumps from green to red in the phase image. These phase jumps are attributed to the limited range of the arctan function from $-\pi/2$ to $\pi/2$. Accordingly, to analyse large phase variations in the measured images it is then necessary to numerically unwrap the phase.

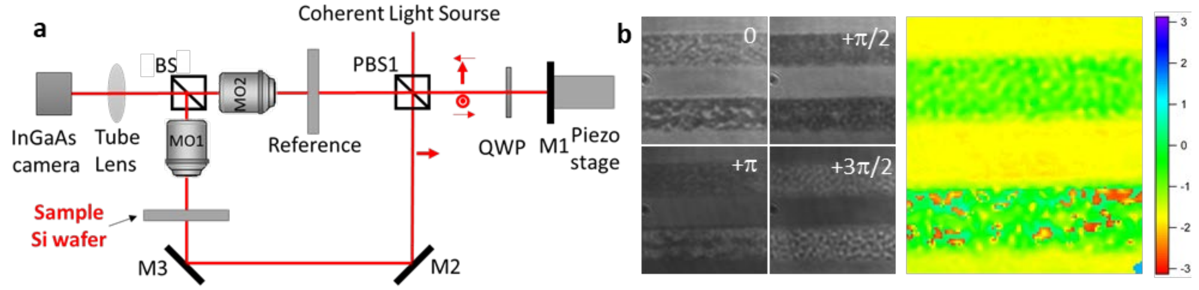

Fig. S8-1. The phase measurement setup. a. Illustration of the measurement setup. **b.** An example of acquisition of the four interference images and the corresponding quantitative phase image numerically retrieved.

8.2 Phase measurements

8.2.1 Single-layer structures

First, we produced and measured phase plates fabricated in a single layer. The number of applied pulses is 10 pulse/μm^2 in all cases. The femtosecond pre-pulse energy is 0.18 μJ to strongly confine the modified layer (see main text). We then change the pulse energy of the picosecond writing pulse to influence on the induced phase delay. Fig.S8-2a shows some results of these experiments. As the picosecond pulse energy decreases, the transmission is improved, and the corresponding phase delay decreases. For comparison, similar experiments are also conducted with pure picosecond pulses with different energy values, as shown in Fig. S8-2b. One important advantage of the new method is the homogeneity of the fabricated structures. This is because the modifications in the latter case develop in the pre-focal region influencing the energy delivery from subsequent pulses.

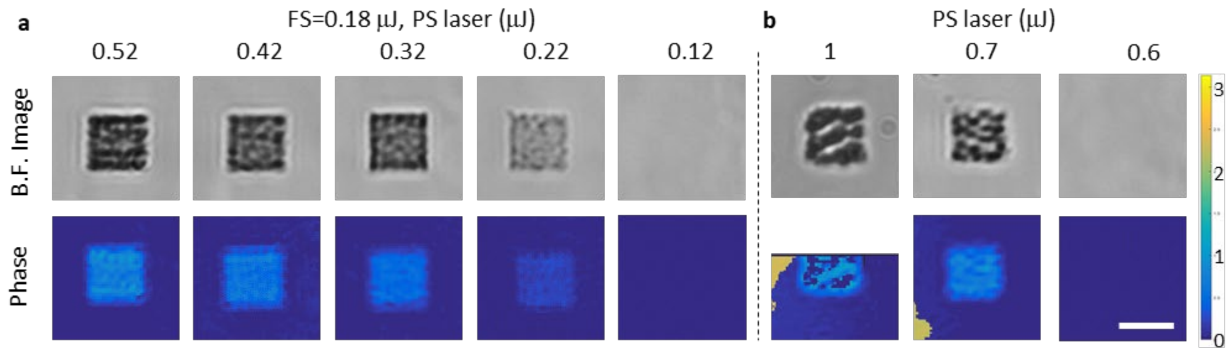

Fig. S8-2. Comparison of the phase objects written by different methods. Bright field (BF) and phase image of the phase plates fabricated using the double-pulse and single-pule approaches with different pulse energies. **a.** Fabricated plates by double-pulse writing, and **b.** by single-pulse writing (pure picosecond case). Scale bar is 20 μm.

In the Fig. S8-3, we compare the phase delay and measured transmission levels for the produced phase plates. The results show a clear trend with a gradual increase of the phase delay with the writing energy. However, the transmission of the phase plates decreases with the increase of the phase delay. Comparing the double-pulse (DP) results and pure picosecond laser writing (PS) results, one notices that the higher energy threshold for modification for pure picosecond laser writing is associated with a minimum phase variation much higher than what can be achieved with the double-pulse configuration. The minimum phase change by DP writing is 0.2 radians while it is around 1 by PS writing for conditions close to modification thresholds. This is mainly attributed to the major difference in writing resolution and modified volumes between the two writing approaches (see main text).

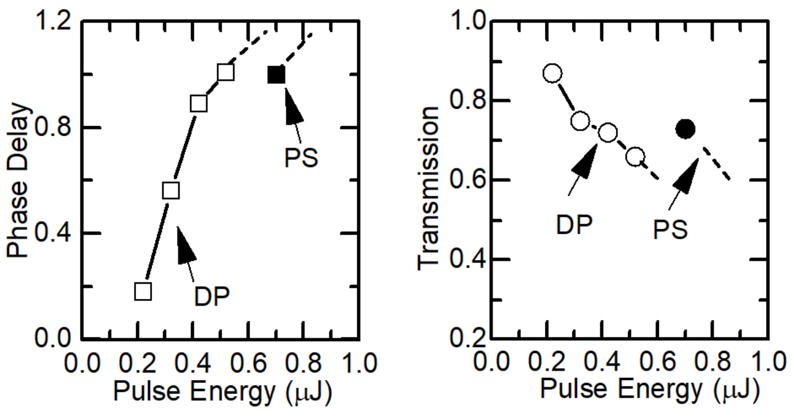

Fig. S8-3. Measured phase delay and transmission through planes modified inside silicon by double-pulse (DP) and pure picosecond laser writing (PS) with different pulse energies.

It is worth noting that the induced phase delay with a single-layer modification is limited to values below 1.2. Writing at higher energies tends to degrade the uniformity of the modifications more than increasing the phase delay. To achieve higher phase delays, we thus worked on multi-layer structures.

8.2.2 Multi-layer structures

We have fabricated phase plates using the high-resolution laser writing conditions (see above, femtosecond pre-pulse of 0.18μJ and picosecond writing pulse of 2μJ) and producing different layers on top of each other inside silicon. The gap between each layer is around 3.5 μm. The measured phase delays for 1 to 6 modified layers are shown in Fig. S8-4a. The following figure shows the phase plate consisting of 1 to 6 layers. From 1 to 3 layers, the phase plate exhibits a relatively good homogeneity. With further increase of the number of layers, inhomogeneities become more apparent even if no obvious disruptions are observed in the fabricated structures. From the phase images, we have retrieved the phase delays and presented the results as a function of the number of layers in Fig. S8-4b. The phase delay is found to be nearly proportional to the number of layers, proving the relevance of the approach.

From these and single-layer experiments, we can conclude that the phase delay can be precisely controlled. The range of the phase delay can be as low as 0.2 radians, and the maximum accessible phase delay can be tuned by increasing the number of modified layers. The large range of accessible phase delays can be directly applied in applications including beam shaping and holography.

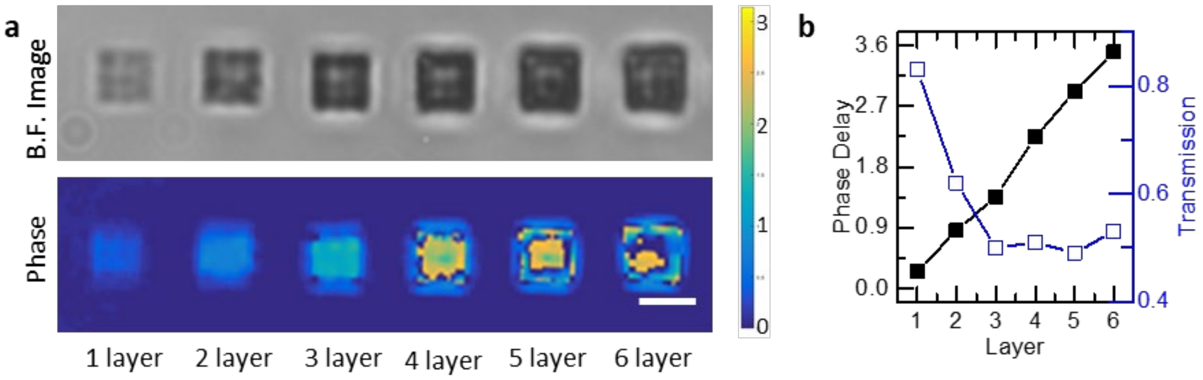

Fig. S8-4. Phase images of modified multiple layers. a. Bright field and phase images of the fabricated phase plates for different number of layers. Phase jumps are observed in the images for 4 to 6 layers because of the measurement of the wrapped phase. Scale bar is 10 μm. **b.** Averaged phase delays and transmission of the corresponding fabricated structures.

8.3 Beam shaping demonstrations

Exploiting the demonstrated level of flexibility for phase control, we have conducted experiments aiming at the fabrication of functional phase plates for beam shaping. In this context, we have first tested the case of a step-phase plate. After validating the functionality and efficiency of the structure, we have investigated more complex phase patterns as shown in the main manuscript.

8.3.1 Measurement setup and simulation

We have fabricated phase plates and investigated the induced beam shaping effects using the setup shown in Fig. S8-5. In the setup (Fig. S8-5a), an incident Gaussian beam (low intensity laser) is focused by a 0.1NA lens. The phase plate is then placed 1 mm before the focused spot. At this position the incident laser beam is smaller than the fabricated structures and so it intercepts efficiently the created phase plates. Then, the shaped focusing spot is measured by a Z-scan imaging system that consists of a

0.7NA (100x) objective lens, a tube lens, and an InGaAs Camera. Based on the imaged beam profiles at different Z, a 3D fluence distribution can be reconstructed. Fig. S8-5b and c show a step-phase plate and the corresponding focal spot as retrieved by the method.

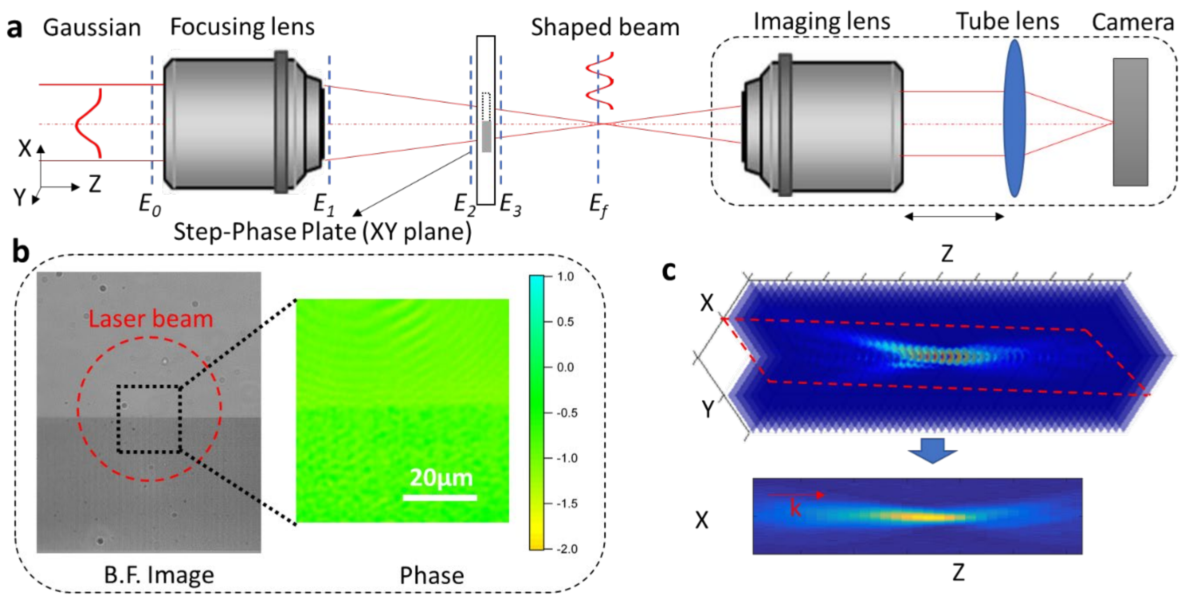

Fig.S8-5. Measurement of the shaping effect of a step-phase plate. a. Simplified schematic of the setup for beam shaping measurements. **b.** Bright field and phase images of the step-phase plate. **c.** 3D intensity distribution and its cross section along XZ plane as retrieved near focus.

To verify the efficiency of the produced phase plates for pulse shaping, we simulate the theoretical intensity distribution using Fresnel diffraction theory[10]. The incident beam is a Gaussian beam under the assumption of a flat wavefront, which can be described as:

$$E_0(x,y) = A_0(x,y)exp(\frac{x^2+y^2}{\omega_0^2}) \quad (8\text{-}3)$$

where the A_0 represents the amplitude of the electric field and ω_0 is the beam waist. The focusing lens can be regarded as a phase element that can be approximated by using the slow varying envelope. After the lens, the electric field can be given by:

$$E_1(x,y) = E_0(x,y)exp(-ik\frac{x^2+y^2}{2f}) \quad (8\text{-}4)$$

where k is the wave vector and the f is the effective focal length of the objective lens. The laser field before the phase plate can be obtained by using the Fresnel diffraction integral:

$$E_2(x,y) = \frac{\exp(ikd_1)}{i\lambda d_1}\iint E_1(x',y')\exp[ik\frac{(x-x')^2+(y-y')^2}{2d_1}]dx'dy' \quad (8\text{-}5)$$

where d_1 represent the distance between the lens and the phase plate, λ is the wavelength. After passing through the phase plate, the electric field becomes:

$$E_3(x,y) = E_2(x,y)\exp(i\Delta\varphi(x,y)) \quad (8\text{-}6)$$

where $\Delta\varphi$ is the phase delay induced by the phase plate. Finally, the electric field near the focusing spot can be calculated again by using the Fresnel diffraction integral:

$$E_f(x,y,z) = \frac{\exp(ikd_2)}{i\lambda(d_2+z)}\iint E_3(x',y')\exp[ik\frac{(x-x')^2+(y-y')^2}{2(d_2+z)}]dx'dy' \quad (8\text{-}7)$$

where d_2 represent the distance between the phase plate and the focusing plane. Combining these equations (3-7), the 3D fluence can be reconstructed accordingly.

8.3.2 Fabrication and characterisation of step-phase plates

To test the applicability of our writing method for phase plate writing, we have fabricated step-phase plates and measured the beam shaping effects using different number of modified layers. The characterization of the phase plate is shown in Fig. S8-5b. The incident laser overlaps with the centre of the step-phase plate. The measured and simulated beam shaping effects are shown in Fig. S8-6. We can clearly see the splitting of the focused spot into two spots with different intensity ratio depending on the apparent phase magnitude for the step-phase plate. The measured results on the left correspond well to the theoretical ones shown on the right assuming a phase level as measured with the quantitative phase infrared microscope (e.g. Fig. S8-5b). This result confirms the functionality and efficiency of the phase plate and so the performance of the laser writing approach for the fabrication of phase elements.

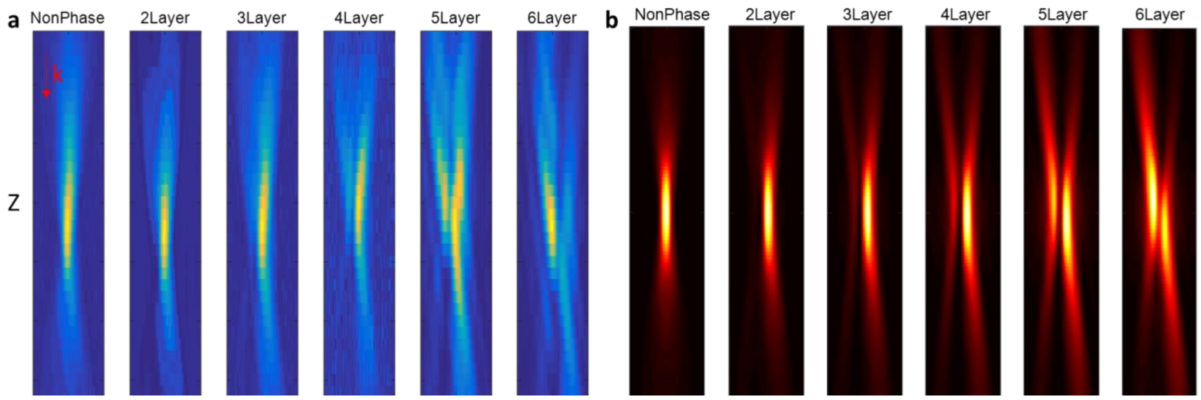

Fig. S8-6. Comparison between simulated and measured focal spots through laser fabricated step-phase plates of different thicknesses inside silicon. a. Measured fluence distributions (cross-section along XZ plane). **b.** Simulated fluence distributions (cross-section along XZ plane) accounting for the insertion of the phase-plates.

References

1. Wang, A., Das, A. & Grojo, D. Temporal-contrast imperfections as drivers for ultrafast laser modifications in bulk silicon. *Phys Rev Res* **2**, 033023 (2020).

2. Das, A., Wang, A., Uteza, O. & Grojo, D. Pulse-duration dependence of laser-induced modifications inside silicon. *Opt Express* **28**, 26623–26635 (2020).

3. Pierce and Spicer. Optical constants of Silicon. (1972).

4. Sokolowski-Tinten, K. & von der Linde, D. Generation of dense electron-hole plasmas in silicon. *Phys Rev B* **61**, 2643 (2000).

5. Mouskeftaras, A. *et al.* Self-limited underdense microplasmas in bulk silicon induced by ultrashort laser pulses. *Appl Phys Lett* **105**, 191103 (2014).

6. Shipilo, D. E. *et al.* Tight focusing of electromagnetic fields by large-aperture mirrors. *Phys Rev E* **100**, 033316 (2019).

7. Kämmer, H. *et al.* Origin of Waveguiding in Ultrashort Pulse Structured Silicon. *Laser Photon Rev* **13**, 1800268 (2019).

8. Mori, M. *et al.* Tailoring thermoelectric properties of nanostructured crystal silicon fabricated by infrared femtosecond laser direct writing. *Physica Status Solidi (A) Applications and Materials Science* **212**, 715–721 (2015).

9. Li, Q., Chambonneau, M., Chanal, M. & Grojo, D. Quantitative-phase microscopy of nanosecond laser-induced micro-modifications inside silicon. *Appl Opt* **55**, 9577 (2016).

10. Wang, A. *et al.* Mask-Free Patterning of High-Conductivity Metal Nanowires in Open Air by Spatially Modulated Femtosecond Laser Pulses. *Advanced Materials* **27**, 6238–6243 (2015).
